# Assessing the effects of N-acetyl cysteine on growth, antioxidant and immune response in tilapia (*Oreochromis niloticus*) under different regimes of stocking densities

**DOI:** 10.1371/journal.pone.0307212

**Published:** 2024-09-30

**Authors:** Wajeeha Komal, Shafaq Fatima, Qandeel Minahal, Razia Liaqat, Aya S. Hussain

**Affiliations:** 1 Department of Zoology, Faculty of Natural Sciences, Lahore College for Women University, Lahore, Punjab, Pakistan; 2 Department of Forestry and Natural Resources, Purdue University, West Lafayette, IN, United States of America; 3 Zoology Department, Faculty of Science, Suez University, Suez, Egypt; Tanta University Faculty of Agriculture, EGYPT

## Abstract

The study investigated the impact of N-acetyl cysteine on growth, immune response, and antioxidant activity in tilapia (*Oreochromis niloticus*). Fish were reared at three densities (1.50, 3.00, and 4.50 kg/m^3^) with four levels of N-acetyl cysteine supplementation (0, 2, 4, and 6 mg/kg) over 60 days. Better growth was observed at low density, but at all densities, fish fed the highest N-acetyl cysteine level (6 mg/kg) showed improved growth. Chemical composition of fish and activity of amylase, lipase and protease in all treatments were noted to be insignificant. The levels of antioxidant enzymes (catalase, superoxide dismutase and glutathione peroxidase) and cortisol in HD treatments were high as compared to LD and MD treatment. However, fish fed with N3 diet in each density treatment showed the lowest level of antioxidant enzymes as well as cortisol. Similarly, the levels of malondialdehyde were noted to be high at HD treatments as compared to that in LD and MD. Its levels were lower in fish fed with N3 diets in all density treatments. Expression of somatostatins-1 did not increase in MD and HD treatments in response to high stocking density when compared with LD treatment. However, pro-opiomelanocortin-α level was reduced after N3 diet in HD treatment and interleukin 1-β expression increased after N3 supplement in HD treatment. In conclusion, N-acetyl cysteine supplementation improved growth and antioxidant response in tilapia. The most optimum dose of N-acetyl cysteine was noted to be 6 mg/kg at high stocking, suggesting the potential role of this nutraceutical in tilapia intensive culture.

## 1. Introduction

Fish is widely recognized as a crucial source of animal protein for people worldwide [1]. There is a pressing need to boost the development of aquaculture to meet the increasing global demand for fish. In 2020, the worldwide production of tilapia reached seven million tonnes [2]. Nile tilapia (*Oreochromis niloticus*) is particularly valued for aquaculture due to its fast growth, minimal reliance on costly animal protein in its diet, and ability to thrive in high stocking densities in intensive production setups. The effectiveness and profitability of intensive fish farming systems rely on the growth rate of the fish and the stocking density utilized [3].

Crowding fish into limited space has detrimental effects on their physiology, leading to reduced growth rates, high levels of cortisol, increased mortality, and oxidative stress. This practice compromises the fish growth performance due to deteriorating water quality, disruptions in social behavior, and alterations in metabolic rates resulting from the stress of overcrowding [4]. These stress responses in fish are closely tied to hormonal reactions in the brain, particularly through the hypothalamic-pituitary interrenal (HPI) axis, which triggers the release of corticotropin-releasing hormone (CRH) in the hypothalamus [5]. Assessing the impact of stocking density on fish physiology often involves examining common parameters such as blood composition and alkaline phosphatase (ALP) levels [6], aspartate aminotransferase (AST) [7], hemoglobin and red blood cells, albumin, globulin and triglycerides [8], immune cells [9]. Studies suggest that high stocking density can adversely affect various blood parameters, including hematology and blood biochemistry [7–9] and can induce chronic stress by elevating cortisol and glucose level [10].

Overcrowding in production systems leads to oxidative stress, which is evident from the increased production of reactive oxygen species (ROS) [11]. This accumulation of free radicals in the form of ROS, happens at a faster rate resulting in various types of cellular damage, such as mutations in DNA [12]. Additionally, oxidative stress impacts the expression of genes associated with energy metabolism (such as peroxisome proliferator-activated receptor gamma coactivator 1-alpha or PGC-1α) [13], growth hormone [14] and insulin-like growth factor-1(IGF-1) [15], myostatin [16]. Other affected factors are related to immunological responses (such as nuclear factor-kβ) [17] and antioxidant enzymes (like nuclear factor erythroid 2-related factor 2 or Nrf2) [18], along with other cellular defense proteins in fish. These biomolecules trigger a cascade of reactions aimed at managing the damage caused by stress.

The study evaluated genetic alterations in fish in response to oxidative stress by examining stress-related genes such as pro-opiomelanocortin-α (POMC-α). Stress responses in fish are triggered by the activation of either the hypothalamic-pituitary-adrenal (HPA) or hypothalamic-pituitary-interrenal (HPI) axis, leading to the release of corticotropin-releasing factor (CRF) and subsequent synthesis of POMC, which is then cleaved into smaller peptides including adrenocorticotropic hormone (ACTH) [19]. ACTH promotes cortisol production, enhancing glucose metabolism to combat stress [20]. Additionally, somatostatin-1 (SST-1) inhibits growth hormone secretion and growth [21, 22], while interleukin-1β (IL-1β) plays a crucial role in immune and inflammatory responses, balancing the immune system and mitigating stress [23].

This oxidative stress can be mitigated by supplementing with natural or synthetic nutraceuticals possessing antioxidant properties like probiotics, probiotics, synbiotics [24], Vitamin C and oxidized fish oil [25]. Recently researchers have found a positive connection between supplementing diets with antioxidants and reducing harmful effects such as health of fish and the activation of stress responses due to stocking density [26]. N-acetylcysteine was not used in response to mitigate oxidative stress against high density. N-acetylcysteine was derived from amino acid cysteine containing thiol, acts as an antioxidant and serves as a precursor for glutathione [27, 28]. N-acetyl cysteine possesses the capability to penetrate cell membranes independently of the amino acid due to the electron gain facilitated by the acetyl portion and is readily soluble in water [29]. One of the primary endogenous defense mechanisms against stress is the glutathione redox cycle [30]. Glutathione (GSH) also functions as a scavenger of free radicals. GSH acts as an electron donor in the reduction of peroxides mediated by glutathione peroxidase (GPx), resulting in the formation of GSH disulfide (GSSG) [31]. GSSG is converted back to GSH with the utilization of NADPH as an electron donor through the action of glutathione reductase (GR). Elevated levels of GSSG and a decreased ratio of GSH/GSSG are considered indicators of oxidative stress. The total intracellular GSH levels decrease during the formation of GSH-S-conjugates by glutathione S-transferases (GST) or through the release of GSH metabolites from cells [31]. De novo synthesis of GSH in all cell types in vivo commences from the constituent amino acids catalyzed by c-glutamylcysteine synthetase (c-GCS), which represents the rate-limiting step in GSH synthesis [32]. c-Glutamyl transpeptidase (c–GT) is the sole enzyme responsible for cleaving the c-glutamyl amide bonds. c-GT initiates the breakdown of extracellular GSH into its constituent amino acids, which can then be transported into the cell [33]. N-acetyl cysteine a well-known thiolic antioxidant, operates through various mechanisms to counter cellular degeneration. It serves as a precursor for GSH synthesis by providing cysteine and stimulates the activity of cytosolic enzymes involved in the GSH cycle such as GR, thereby enhancing the rate of GSH regeneration [34]. N-acetyl cysteine also shields the cell from oxidative damage through direct interaction between its reducing thiol group and reactive oxygen species (ROS) such as hypochlorous acid, hydroxyl radical (OH•), hydrogen peroxide (H_2_O_2_), superoxide anion (O^2^-), peroxynitrite (ONOO^-^), nitrogen dioxide (NO_2_) and (HOX) [35, 36]. It also contributes to the disruption of disulfide bonds and the restoration of thiol pools, which in turn regulate the redox state [37].

The present study investigated the effects of varying doses of N-acetyl cysteine under different stocking density conditions in tilapia. Additionally, the study aimed to identify the optimal N-acetyl cysteine dose required to mitigate the effects of different stocking densities. The objective was to evaluate the influence of a N-acetyl cysteine formulation in the diet on growth performance, stress physiology, antioxidant status, and the expression of genes associated with growth performance (SST-1), stress response (POMC-α) and immune function (IL-1β) in tilapia exposed to varying stocking densities. This optimal dose of N-acetyl cysteine may serve as a dietary supplement in commercial tilapia culture to improve growth and enhance immune response, particularly under high stocking density conditions.

## 2. Materials and methods

### 2.1. Diet preparation

In present study, commercial N-Acetyl Cysteine (C5H9NO3S, Sigma Aldrich, USA; purity ≥ 99%) was used as a feed supplement. The solubility test for NAC in 100mg/ml was clear which indicates that the NAC completely dissolved. Treatment diets were prepared by mixing the finely ground ingredients (Table 1) with four different levels of N-Acetyl Cysteine (N) solutions (N0 = 0 mg/kg, N1 = 2mg/kg, N2 = 4mg/kg, N3 = 6mg/kg). Feed pellets of 1mm were prepared using a mechanical pellet machine (PCSIR Laboratories, Pakistan) (Table 1). The pellets were air-dried at room temperature and stored at 4°C in sealed bags. Fish were fed at a proportion of 2% of their body mass on daily basis, twice a day.

**Table 1:** Feed formulation with N-acetyl cysteine supplementation.

Ingredients (%)	N0	N1	N2	N3
Corn meal	28.00	28.00	28.00	28.00
Rice polish	12.00	12.00	12.00	12.00
Wheat bran	9.00	9.00	9.00	9.00
Canola meal	8.00	8.00	8.00	8.00
Soybean meal	36.00	36.00	36.00	36.00
Dicalcium phosphate	4.00	4.00	4.00	4.00
Methionine	0.79	0.79	0.79	0.79
Lysine	1.32	1.32	1.32	1.32
L-Threonine	0.89	0.89	0.89	0.89
N-Acetyl Cysteine	0.00	0.2	0.4	0.6

### 2.2. Experimental design

**Fig 1:**
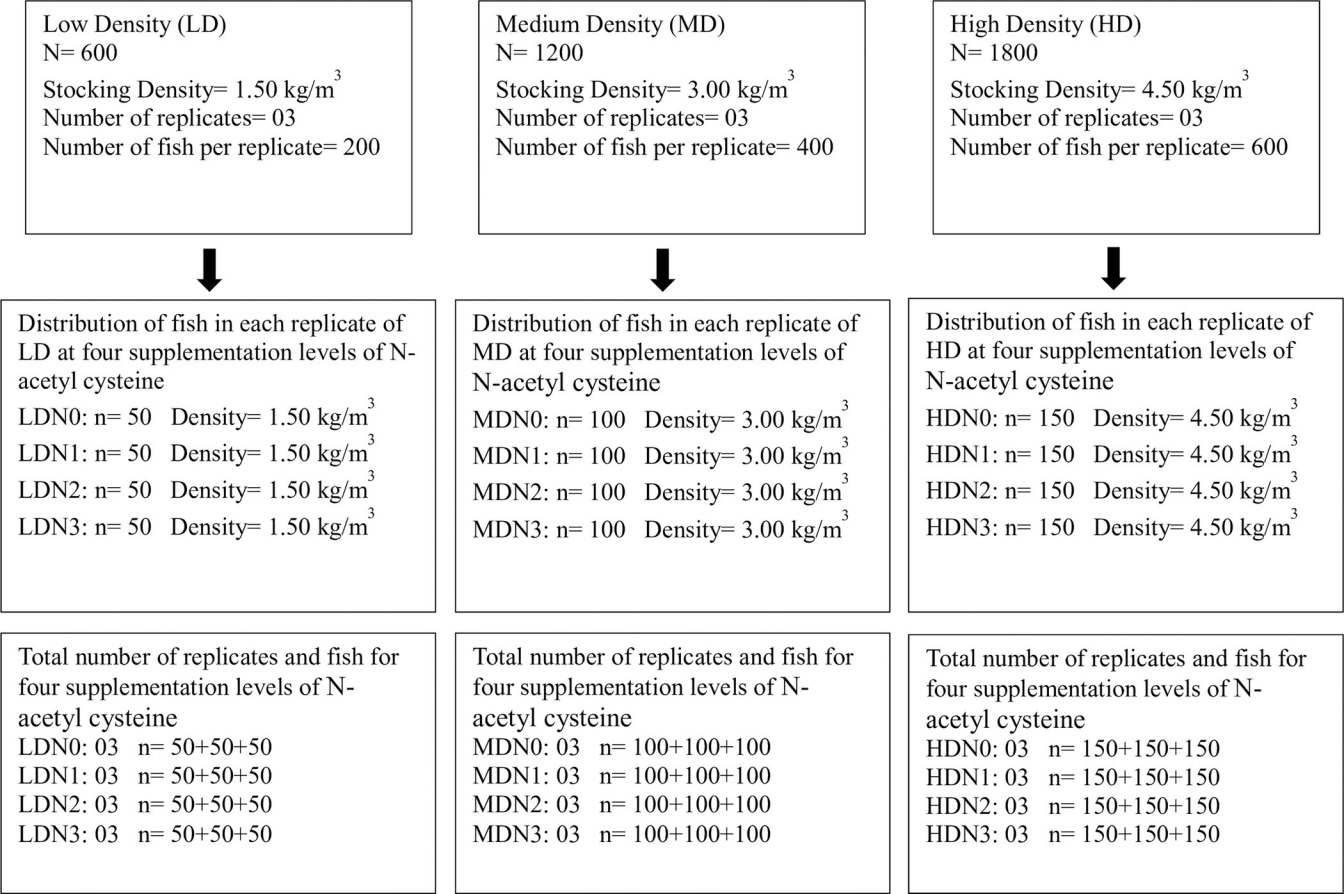
Distribution of fish (initial weight = 80.00±1.20g) in experimental design having three stocking densities (LD, MD, HD) and their replicates at four different level of N-acetyl cysteine supplementation (N0, N1, N2, N3).

The experimental model used for the study was GIFT tilapia which was imported from Thailand in 2012. Tilapia (*Oreochromis niloticus*) (n = 3600, initial weight = 80.00±1.20g) were procured from a local fish hatchery (Lahore, Pakistan) and transferred to Aquaculture facility, Lahore College for Women University. There was no mortality of fish during the transfer. This study was commenced after the approval of Animal Ethics Committee of Department of Zoology, Lahore College for Women University (Approval #: Zoo/LCWU/932). Fish (n = 3600) were randomly distributed in 36 fiber glass tanks (water volume/tank = 1 m^3^) (Fig 1). Water utilized in this study was drawn from an underground source. Each tank has water supplied from the same water sump, treated with UV filter and biofilters. All tanks had their own water supply. Fish were acclimatized for one week before commencement of trial. The duration of this trial was 60 days and all fish were healthy by end of this trial. This time period for the trial was selected following [38], which investigated the growth of tilapia over a period of 171 days, aiming to achieve a final stocking density of 57.81 kg/m^3^. Furthermore, 60 days is the most recommended study period to observe the effect of any dietary supplement on the growth of fish. Duration of trial (60 days) was the only designated humane end point in this study to terminate the experiment. Fish husbandry conditions and health were excellently maintained throughout the entire trial period to minimize the mortality as mentioned in section 2.3 of Methods.

Three stocking density regimes were studied in this trial; low density (LD) (1.50 kg/m^3^), medium density (MD) (3.00 kg/m^3^) and high density (HD) (4.50 kg/m^3^). The total number of fish stocked in LD, MD and HD treatment was 600, 1,200 and 1,800, respectively. Each density treatment had three replicates (Fig 1). Fish in all stocking density treatments (LD, MD, HD) were fed with four different levels of dietary supplementation of N-acetyl cysteine (N0, N1, N2, N3). Dose of each dietary level is given in section 2.1 of Methods. Each N-acetyl cysteine supplementation level was studied in further three replicates (Fig 1). These four different levels of N-acetyl cysteine were fed to low density treatment (LDN0, LDN1, LDN2, LDN3), medium density treatment (MDN0, MDN1, MDN2, MDN3), and high density treatment (HDN0, HDN1, HDN2, HDN3). Fish were fed by hand with daily ration calculated at the rate of 2% of biomass in each replicate. Random weight check of fish was performed for each replicate after every 15 days to adjust the daily ration.

### 2.3. Water quality parameters and survival rate

Water quality was very well maintained in all tanks to ensure the welfare of fish. A total 20% of water was exchanged every day from each tank. Water quality parameters were measured twice a day to ensure its standard quality levels throughout the trial period. Aeration pumps (120V/60Hz, Airmax SilentAir LR25, USA) were used to deliver air via diffuser grids. There was one rectangular diffuser grid in each tank (L ×W: 1 × 0.5 ft). Each diffuser grid was built by using the anti-microbial tubing (outer diameter: 25.4 mm; inner diameter: 12.7 mm; airflow: 2.2 m3/h/meter) to generate microbubbles ensuring the good air saturation in water (>80%). All tanks were back washed on daily basis to drain solid waste settled at the bottom of tank. Water quality parameters including water temperature (25.86 ± 0.30–27.78 ± 0.23°C), dissolved oxygen (4.13 ± 0.31–4.88 ± 0.27 mg/L), and pH (7.52 ± 0.45–8.76 ± 0.01) were monitored twice a day by using portable meters (HI98494, Hanna, USA). Ammonia (0.80±0.01–1.50±0.22 ppm), and nitrite (0.11±0.01–0.21±0.10 mg/L) were monitored twice a week by using commercial kits (HI733, HI93708, Hanna, USA). Fish in every tank were checked for any sign of disease, abnormal behavior and mortality twice a day. Dead fish were removed immediately if found and carefully recorded. Survival rate observed in LD, MD and HD were 100%, 100% and 99.20%, respectively due to well-maintained husbandry conditions over the study period. Mortality of only 0.80% observed in HD treatment, was due to high density. However, it was much lower than the designated limit of 10% mortality, approved by Animal Ethics Committee for Aquaculture trials.

### 2.4. Sample analysis

At end of the trial, five fish were randomly sampled from each replicate (Fig 1) of all density treatments (20 samples per treatments). A total of 180 fish were used for this terminal sampling out of 3600 fish used in the trial following the designated limit of 5% of population for euthanization by Animal Ethics Committee. Remaining 3420 fish were humanely released in nearby lake, administered by Department of Fisheries, Pakistan for stocking purpose (Release Approval #: DOF/27856/2022). Before sampling, fish were fasted for 24 hours. On sampling day, they were euthanized using clove oil (0.8 ml/L of water, Sigma-Aldrich, USA). This dose of clove oil is standard to euthanize fish in a very humane way which took less than ten minutes to euthanize sampled fish.

Blood was collected from the caudal vein in two tubes. One tube was coated with ethylenediamine tetra acetic acid (EDTA: (for hematology) while second tube contained clot activator for plasma collection. Blood samples were centrifuged at 5,000 rpm for 15 minutes and plasma was collected in separate eppendorf tubes and stored at -20°C. The total body weight and total body length were measured before dissection. Fish were dissected, and gills were collected, rinsed in deionized water and preserved in 10% formaldehyde solution for 24 hours for histological study. Fish muscle samples were collected and stored at– 20°C for chemical composition, fatty acids and amino acids analysis following the guidelines of Association of Official Analytical Chemists (AOAC) [39]. Muscle samples were dehydrated in an oven at 80°C until a consistent dry weight was reached. These dried samples were processed for further chemical analysis. The Kjeldahl apparatus (PCSIR Laboratories, Pakistan) was used to determine the crude protein, while crude lipids were identified using the Folch method [40] using the Soxhlet apparatus (PCSIR Laboratories, Pakistan). The ash content in muscles was determined using muffle furnace (PCSIR Laboratories, Pakistan). For quantifying the amino acid contents of fish muscles, an amino acid analyzer (Biochrome 30+, Biochrome Limited, Cambridge, UK) was used, and the analytical procedures were in accordance with those outlined by [11]. The intestinal samples from midgut were weighed, rinsed with deionized water and homogenized in 0.86% sterile normal saline solution (1:9). This mixture was centrifuged at 5000 rpm for 15 minutes. Supernatant was collected and stored at -20°C. Each sample was analyzed in three replicates for each analysis. Liver samples were collected and homogenized in liquid nitrogen at -80°C for genes expression analysis. Condition factor (K), specific growth rate (%) (SGR), hepatosomatic index (HSI), fish weight gain, survival rate, feed conversion rate (FCR) was measured by using the given formulae.


$$Condition\;factor\;(\%)=\frac{Total\;body\;weight}{Total\;body\;{length}^{3}}\times 100$$

$$Specific\;growth\;rate\;(\%)=\frac{Ln\;final\;weight-Ln\;initial\;weight}{Time\;interval\;in\;days}\times 100$$

$$Hepatosomatic\;index=\frac{Liver\;weight}{Total\;body\;weight}\times 100$$

$$Feed\;conversion\;ratio=\frac{Weight\;of\;feed\;consumed}{Weight\;gain\;(wet\;weight)}$$

$$Survival\;rate\;(\%)=\frac{Final\;number\;of\;fish}{Initial\;stocking\;density}\times 100$$

### 2.5. Hematological analysis

Hematological parameters such as hemoglobin (Hb) (g/dl), white blood cells (WBC) (10^3^/μL) count such as Neutrophils (%), Eosinophils (%), Lymphocytes (%), Monocytes (%), red blood cell (RBC) (10^6^/μL) count, platelets (10^3^/μL) were determined by using auto-hematology blood analyzer (Sysmex-KX-21, Japan), calibrated for fish.

### 2.6. Biochemical analysis

Triglyceride (TG) (mg/dl) was estimated through a triglyceride colorimetric assay kit (Thermo Fisher Scientific, USA, CAT No. EEA028) following the protocol of the manufacturer. The level of albumin (Alb) (g/dl) was determined through the use of bromocresol green (BCG) dye binding technique, utilizing an albumin kit (LOT. DR379E249; ANMOL-LAB Pvt. Ltd, India). The quantification of alkaline phosphatase (ALP) (U/L) was carried out using commercial kit (Thermo Fisher Scientific, USA, CAT No. EEA002, E.C. 3. I. 3.1.). Aspartate aminotransferase (AST) (U/L) was estimated through commercial ELISA kit (Thermo Fisher Scientific, USA, CAT No. MAK055, E.C. 2.6. 1.1.). Activity of alanine aminotransferase (ALT) (U/L) was measured using commercial ELISA kit (Thermo Fisher Scientific, USA, CAT No. MAK052, E.C. 2.6. 1.2.). The concentration of glucose (GLU) (mg/dl) was measured by using laboratory blood glucose analyser (Human, Germany).

### 2.7. Cortisol assay

The concentration of cortisol (ng/ml) in blood plasma was measured using ELISA (Calbiotech, USA, CAT No. CO368S, CID 5754) having a sensitivity of 1.16 ng/ml. The intra-assay and inter-assay precision were 3.8% and 8.65%, respectively. The detection limit was 0–800 ng/ml. The absorbance value was read on spectrophotometer at 450 nm.

### 2.8. Antioxidants assay

Plasma catalase (CAT) (U/ml) activity was determined using a commercial ELISA colorimetric activity kit (Thermo Fisher Scientific, USA, CAT No. EIACATC, *EC 1*.*11*.*1*.*6*) having an analytical sensitivity of 0.052 U/ml. The absorbance was read at 560 nm at 37°C. The activity of superoxide dismutase (SOD) (ng/ml) were measured by using ELISA kit (PARS BIOCHME, China, CAT No. PRS-02005hu, EC 1.15.1.1) with an assay range of 0.3 ng/ml– 10 ng/ml. Malondialdehyde (MDA) (nmol/ml) ELISA kit (PARS BIOCHME, China, CAT No. PRS-00991hu, CAS 542-78-9) with an assay range of 0.3 ng/ml– 7 nmol/ml. Activity of glutathione peroxidase (GPx) (IU/ml) were measured by using ELISA kit (PARS BIOCHME, China, CAT No. PRS-00680hu, EC 1.11. 1.9) with an assay range of 3 IU/ml– 200 IU/ml. The absorbance value of SOD, MDA, and GPx was read at 450 nm and 37°C.

### 2.9. Digestive enzymes assay

For digestive enzyme analyses, the supernatant of processed whole intestine samples was utilized. Activity of lipase (U/L) was assayed with a commercial ELISA kit (Sigma Aldrich, USA, CAT No. MAK046, EC 3.1.1.3) with a detection limit of 5 U/L to 250 U/L at 37°C and 570 nm of wavelength. Amylase (U/L) activity was measured using a commercial ELISA kit (Sigma Aldrich, USA, CAT No. *MAK009A*, EC 3.2. 1.1.) with a detection limit of 0.2439 U/L—2200 U/L at 37°C and 405 nm of wavelength. The activity of protease was determined following instructions of Walter, 1984. Casein 1% w/v was used as substrate in 0.2 M phosphate buffer at pH 7.0. One unit of protease indicates the amount of enzyme that releases 1 μg/ml/min of tyrosine determined at 660 nm of wavelength.

### 2.10. Histological study

Preserved gill samples were dehydrated by passing through different grades of alcohol (70%, 90% and 100%) and xylene. For the infiltration of wax, gills were processed in paraffin wax. Microtome were used for sectioning and wax blocks were trimmed at 10 μ and then transverse sections of 4 μ thickness were cut. For dewaxing, xylene and alcohol were used and stained with haematoxyline and eosin. Stained section of gills was mounted with DPX (mixture of distyrene, plasticizer and xylene) (Merck, Germany). Microphotographs were taken at digital camera fitted optical microscope (Trinocular E-200, Nikon Japan Eil-12). Histological analysis of gills was performed to determine the alteration in lamella structure including primary and secondary or any other disruption including necrosis (tissue death), epithelial lifting (detachment of epithelial cells) and blood congestion (accumulation of blood).

### 2.11. Gene expression analysis

Liver samples (50 mg/sample) were used to extract total RNA by using trizol (Catalog No. 15596026, Thermo, USA) method at 37°C. The quality and quantity of each sample was verified on Nanodrop 2000 spectrophotometer (Thermo, Waltham, MA, USA). The first strand cDNA was synthesized using super script III first strand cDNA synthesis kit (Cat No. 18080051, Life technologies). The 5.0 μg of total RNA was used for cDNA synthesis. cDNA synthesis was performed in the first step with poly-A tail primedoligodT in a total volume of 20 μl. The first reaction mixture was prepared having RNA 5 μg, 50 μMoligo (dT) 20 of 1 μL, 10 mMdNTP mix of 1 μL and then water was added upto 10 μL. The mixture was incubated at 65°C for 5 min. cDNA synthesis Mix-2 was prepared by adding 10 X RT buffer (2 μL), 25 mM MgCl_2_ (4 μL), 0.1 M DTT (2 μL), RNaseOUT™ (40 U/μL) (1 μL), SuperScript® III RT (200 U/μL) (1 μL), a total of 10 μL.

The prepared 10 μL of cDNA Synthesis mix was added to each RNA/primer mixture, mixed gently and collected by brief centrifugation. The tube was incubated for 50 min at 50°C. The reaction was terminated at 85°C for 5 min. The cDNA was store at -20°C. The PCR reaction was performed in a separate tube with gene specific primers (forward and reverse) using 2 μl cDNA templates. The following set of primer was used for real-time PCR which were designed by using software Primer Quest from integrated DNA technology (Table 2). Each set of primer 1 μl (10 μM) along with 12.5 μl SYBR green PCR master mix (Maxima SYBR Green/ROX qPCR Master Mix (2X)) were used. First denaturation step was carried out as 95°C for 2 min, followed by 95°C denaturation for 15 sec, annealing step was carried out at 55°C for 1 min and extension step was carried out at 72°C for 1 min. β-actin was used as the housekeeping gene for reference. The 2-fold induction was determined by ΔΔCT method (relative quantification).

**Table 2:** Primer sequence of genes.

#	Gene	Sequence 5 to 3
01	Somatostatin-1 (SST-1) F	TGCTGGGCTCCAAACAG
02	Somatostatin-1 (SST-1) R	AGGGAAGTTCTCCTCTTCCA
03	Interleukin 1-β (IL-1β) F	TGGAGGAGGTGACGGATAAA
04	Interleukin 1-β (IL-1β) R	CAGTGTCGCGTTTGTAGAAGA
05	Proopiomelanocortin (POMC-α) F	CTCCTACTCAATGGAGCACTTC
06	Proopiomelanocortin (POMC-α) R	AAGCTCTCGTCTCCTCATCT
07	β-Actin-F	GAGGTATCCTGACCCTGAAGTA
08	Β-Actin-R	ACTCTCAGCTCGTTGTAGGA

### 2.12. Statistical analysis

For all the statistical analyses, SPSS v.29 software was used. Data were presented as Mean± SE for all the parameters. Kolmogorov–Smirnov test was performed to assess the normality of data. Levene test were performed to check the homogeneity of variance of data. The effect of density and N-acetyl cysteine supplementation on different parameters was determined by Two-Way ANOVA. To reject the null hypothesis, 0.05 probability level was used.

## 3. Results

### 3.1. Growth

In three different density treatments (LDN, MDN, HDN), total body length (df_2_, F = 42.74), total body weight (df_2_, F = 756.21), condition factor (df_2_, F = 223.82), specific growth rate (df_2_, F = 606.34), hepatosomatic index (df_2_, F = 0.630) were found to be significantly (P≤ 0.05) different (Table 3). An insignificant variation (P≥ 0.05) in total body length (df_3_, F = 2.79), total body weight (df_3_, F = 0.65), condition factor (df_3,_ F = 1.59) and specific growth rate (df_3,_ F = 0.14) were observed except hepatosomatic index (df_3,_ F = 8.25) (P≤ 0.05) were observed between different levels of N-acetyl cysteine (N) supplementation in all three-density treatment (levels of supplementation: 04 in each treatment) except in total body length and hepatosomatic index. Other than this, effect of density* N-acetyl cysteine calculated by two-way ANOVA also showed a significant effect (P≤ 0.05) on total body length (df_6_, F = 14.46), total body weight (df_6_, F = 13.56), condition factor (df_6_, F = 9.85), specific growth rate (df_6_, F = 12.48) and hepatosomatic index (df_6_, F = 9.77). The survival rate of fish both in LSD and MSD was 100% but in HSD the its rate was 100 ±0.05% - 99.20 ±0.06%.

**Table 3:** Analysis of total body length (cm), total body weight (g), condition factor, hepatosomatic index, specific growth rate and FCR (Mean ± SE) in three density treatment (LDN (1.50 kg/m^3^), MDN (3.00 kg/m^3^), HDN (4.50 kg/m^3^)) having four N-acetyl cysteine supplementation levels (N0 = 0 mg/kg, N1 = 02 mg/kg, N2 = 04 mg/kg, N3 = 06 mg/kg).

Treatments	TBL (cm)	TBW (g)	SGR (%)	K (%)	HSI (%)	FCR	Survival rate (%)
**LDN0**	21.50±0.28	207.25±1.20	518.85±1.24	2.11±0.29	1.05±0.03	0.75±0.39	100.00±0.01
**LDN1**	20.00±0.39	201.50±1.99	516.04±1.34	2.53±0.55	1.03±0.07	0.79±0.44	100.00±0.01
**LDN2**	18.58±0.44	192.25±1.11	511.30±1.62	3.08±0.76	0.96±0.05	0.86±0.43	100.00±0.02
**LDN3**	19.25±0.54	194.50±1.13	512.48±1.86	2.75±0.54	1.00±0.03	0.84±0.43	100.00±0.03
**MDN0**	15.50±0.24	160.25±1.23	493.09±1.40	4.41±0.57	0.97±0.02	1.20±0.54	100.00±0.04
**MDN1**	14.88±0.38	150.00±1.32	486.08±1.54	4.71±0.54	0.98±0.04	1.37±0.64	100.00±0.02
**MDN2**	15.13±0.39	152.50±1.07	487.93±1.60	4.50±0.30	0.98±0.03	1.32±0.43	100.00±0.03
**MDN3**	15.38±0.44	156.00±1.88	490.31±1.33	4.37±0.46	0.96±0.03	1.26±0.29	100.00±0.05
**HDN0**	13.50±0.33	136.25±1.24	476.86±1.43	5.56±0.39	0.98±0.05	1.71±0.39	99.20±0.06
**HDN1**	15.25±0.39	148.75±1.66	485.58±1.55	4.23±0.67	1.31±0.04	1.40±0.44	100.00±0.08
**HDN2**	15.00±0.24	152.00±1.69	487.69±1.40	4.61±0.78	1.35±0.03	1.33±0.43	100.00±0.09
**HDN3**	15.00±0.50	149.50±1.59	485.78±1.47	3.09±0.40	1.02±0.05	1.38±0.32	100.00±0.05

### 3.2. Chemical composition of muscles

The moisture content (df_2_, F = 231525.00), crude protein (df_2_, F = 5308.33), crude ash (df_2_, F = 0.05) and crude fat (df_2_, F = 3172.75) was significantly different (P≤ 0.05) between three density treatment (Table 4). A significant difference (P≤ 0.05) in the content of moisture (df_3_, F = 41922.22), crude protein (df_3_, F = 4822.22), crude ash (df_3_, F = 2292.11) and crude fat (df_3_, F = 3396.36) was observed between different levels of N-acetyl cysteine supplementation in all three-density treatment (levels of supplementation: 04 in each treatment). Effect of density*N-acetyl cysteine also showed significant effect (P≤ 0.05) on moisture (df_6_, F = 40580.55), crude protein (df_6_, F = 2663.88), crude ash (df_6_, F = 2012.11) and crude fat (df_6_, F = 4580.52).

**Table 4:** Analysis (Mean±SE) of chemical composition (%) of muscles in three density treatment (LDN (1.50 kg/m^3^), MDN (3.00 kg/m^3^), HDN (4.50 kg/m^3^)) having four N-acetyl cysteine supplementation levels (N0 = 0 mg/kg, N1 = 02 mg/kg, N2 = 04 mg/kg, N3 = 06 mg/kg).

Groups	Moisture (%)	Crude protein (%)	Crude fat (%)	Crude ash (%)
**LDN0**	73.80±0.33	18.50±0.32	2.10±0.43	5.60±0.60
**LDN1**	74.10±0.45	18.30±0.23	1.90±0.54	5.70±0.65
**LDN2**	73.90±0.54	18.60±0.32	1.87±0.65	5.54±0.54
**LDN3**	74.30±0.35	18.90±0.42	1.15±0.45	5.65±0.45
**MDN0**	74.12±0.23	18.38±0.34	1.70±0.54	5.80±0.55
**MDN1**	74.20±076	18.70±0.23	1.60±0.34	5.50±0.46
**MDN2**	74.15±0.76	18.75±0.34	1.90±0.45	5.20±0.44
**MDN3**	74.03±0.60	18.50±0.54	1.75±0.43	5.72±0.56
**HDN0**	74.30±0.65	18.45±0.43	1.52±0.76	5.73±0.65
**HDN1**	74.00±0.65	18.90±0.33	1.54±0.34	5.56±0.35
**HDN2**	73.80±0.55	18.92±0.44	1.68±0.65	5.60±0.75
**HDN3**	74.04±0.50	18.51±0.43	1.63±0.44	5.87±0.76

### 3.3. Profile of amino acids

A significant difference (P≤ 0.05) has been observed (Table 5) in the profile of amino acid methionine (df_2_, F = 48260.33), threonine (df_2_, F = 74393.08), valine (df_2_, F = 17791.08), isoleucine (df_2_, F = 3498.25), leucine (df_2_, F = 65941.75), phenylalanine (df_2_, F = 48294.75), histidine (df_2_, F = 14013.12), lysine (df_2_, F = 100579.79), arginine (df_2_, F = 30533.82), ornithine (df_2_, F = 2719.64), cysteine (df_2_, F = 3425.70), aspartic acid (df_2_, F = 319463.29), asparagine (df_2_, F = 136454.20), serine (df_2_, F = 318326.03), glutamic acid (df_2_, F = 400357.27), glycine (df_2_, F = 46067.76), alanine (df_2_, F = 42082.31), proline (df_2_, F = 13059.78) and tyrosine (df_2_, F = 24369.72) among three different densities treatment (LDN, MDN, HDN). Profile of amino acids including methionine (df_3_, F = 83931.55), threonine (df_3_, F = 36966.22), valine (df_3_, F = 30605.55), isoleucine (df_3_, F = 214863.44), leucine (df_3_, F = 60669.77), phenylalanine (df_3_, F = 37662.13), histidine (df_3_, F = 53613.23), lysine (df_3_, F = 37540.51), arginine (df_3_, F = 308163.73), ornithine (df_3_, F = 1834.63), cysteine (df_3_, F = 1247.63), aspartic acid (df_3_, F = 6026.99), asparagine (df_3_, F = 1179089.23), serine (df_3_, F = 47595.71), glutamic acid (df_3_, F = 817760.86), glycine (df_3_, F = 38551.57), alanine (df_3_, F = 16615.81), proline (df_3_, F = 138633.64) and tyrosine (df_3_, F = 1526.40) showed a significant difference (P≤ 0.05) between different levels of N-acetyl cysteine (N) supplementation in all three-density treatment. Effect of density* N-acetyl cysteine also showed a significant effect (P≤ 0.05) on methionine (df_6_, F = 50674.55), threonine (df_6_, F = 89303.30), valine (df_6_, F = 25282.63), isoleucine (df_6_, F = 43989.36), leucine (df_6_, F = 148859.86), phenylalanine (df_6_, F = 77551.63), histidine (df_6_, F = 34756.70), lysine (df_6_, F = 186572.40), arginine (df_6_, F = 174546.99), ornithine (df_6_, F = 4143.83), cysteine (df_6_, F = 4775.87), aspartic acid (df_6_, F = 456403.48), asparagine (df_6_, F = 437713.15), serine (df_6_, F = 537234.83), glutamic acid (df_6_, F = 990334.47), glycine (df_6_, F = 171558.97), alanine (df_6_, F = 585479.66), proline (df_6_, F = 75450.29) and tyrosine (df_3_, F = 108387.59).

**Table 5:** Analysis (Mean±SE) of amino acids (%) of muscles in three density treatment (LDN (1.50 kg/m^3^), MDN (3.00 kg/m^3^), HDN (4.50 kg/m^3^)) having four N-acetyl cysteine supplementation levels (N0 = 0 mg/kg, N1 = 02 mg/kg, N2 = 04 mg/kg, N3 = 06 mg/kg). Values are expressed as mg of amino acid per g of crude protein (mg/gcp).

**Essential Amino Acids**																	
**Treatments**	**Methionine**		**Threonine**		**Valine**		**Isoleucine**		**Leucine**		**Phenylalanine**		**Histidine**	**Lysine**		**Arginine**	**Ornithine**
**LDN0**	21.46±0.43		36.55±0.55		37.11±0.56		34.18±0.65		60.57±0.64		33.56±0.54		22.68±0.65	56.46±0.54		49.69±0.54	2.34±0.54
**LDN1**	22.80±0.43		38.58±0.32		37.12±0.65		34.68±0.44		61.59±0.53		33.35±0.54		22.26±0.45	59.35±0.56		49.60±0.56	2.23±0.43
**LDN2**	22.58±0.46		37.15±0.43		37.36±0.44		33.60±0.33		59.60±0.64		32.57±0.34		21.81±0.43	57.67±0.44		48.56±0.76	2.53±0.65
**LDN3**	23.58±0.66		36.57±0.23		36.36±0.54		35.47±0.22		58.68±0.51		34.57±0.34		22.80±0.32	56.66±0.45		51.59±0.55	2.13±0.43
**MDN0**	23.23±0.45		38.03±0.12		36.56±0.65		32.44±0.55		58.70±0.48		31.76±0.23		20.67±0.34	55.23±0.45		49.24±0.45	2.69±0.34
**MDN1**	24.46±0.66		39.22±0.45		35.69±0.75		35.75±0.60		61.55±0.66		32.36±0.34		22.69±0.54	55.82±0.34		52.91±0.34	2.80±0.46
**MDN2**	21.58±0.54		37.03±0.65		37.24±0.65		34.24±0.65		60.81±0.76		33.46±0.23		22.26±0.34	56.26±0.45		49.14±0.23	2.26±0.64
**MDN3**	22.89±0.55		38.47±0.55		37.04±0.54		34.61±0.62		61.89±0.74		33.32±0.34		22.18±0.43	59.18±0.65		48.71±0.56	2.18±0.54
**HDN0**	22.67±0.76		37.23±0.66		37.47±0.65		33.68±0.65		59.79±0.64		32.78±0.34		21.89±0.45	57.85±0.34		48.43±0.56	2.55±0.34
**HDN1**	23.36±0.44		36.79±0.76		36.24±0.54		35.24±0.75		58.82±0.64		34.60±0.23		22.71±0.65	56.37±0.35		51.26±0.23	2.26±0.42
**HDN2**	23.36±0.55		38.79±0.55		36.48±0.54		32.80±0.63		59.01±0.74		31.93±0.54		20.91±0.45	55.45±0.35		49.35±0.45	2.48±0.44
**HDN3**	24.35±0.45		39.15±0.76		35.81±0.65		35.69±0.54		61.51±0.54		32.25±0.45		22.82±0.76	55.93±0.56		52.92±0.65	2.59±0.42
**Non-Essential Amino Acids**																	
**Treatments**		**Cysteine**		**Aspartic Acid**	**Asparagine**	**Serine**		**Glutamic acid**		**Glycine**		**Alanine**	**Proline**		**Tyrosine**		
**LDN0**		8.71±0.43		63.79±0.43	56.11±0.43	36.53±0.54		112.55±0.62		55.21±0.55		58.78±0.72	39.13±0.54		20.33±0.26		
**LDN1**		8.55±0.23		63.59±0.32	55.81±0.32	35.70±0.46		109.87±0.45		52.85±0.65		55.84±0.70	39.75±0.54		18.82±0.23		
**LDN2**		8.63±0.12		63.64±0.34	55.27±0.34	36.48±0.46		111.80±0.54		54.74±0.73		58.63±0.51	39.47±0.65		20.59±0.23		
**LDN3**		8.11±0.21		60.91±0.45	60.47±0.34	31.83±0.45		118.73±0.61		52.85±0.61		54.89±0.52	39.71±0.64		19.85±0.24		
**MDN0**		8.10±0.22		65.37±0.54	54.48±0.56	30.51±0.83		108.61±0.34		51.63±0.61		55.65±0.41	38.76±0.76		19.31±0.25		
**MDN1**		8.49±0.32		67.45±0.44	61.45±0.45	33.46±0.64		114.58±0.43		53.50±0.52		60.68±0.55	41.88±0.65		21.40±0.24		
**MDN2**		8.23±0.12		63.61±0.45	56.04±0.54	34.61±0.54		112.47±0.64		55.18±0.48		58.90±0.66	39.04±0.55		20.33±0.29		
**MDN3**		8.43±0.23		64.00±0.54	55.92±0.34	35.49±0.43		109.78±0.63		52.92±0.51		56.01±0.77	39.73±0.76		18.93±0.26		
**HDN0**		8.01±0.12		63.42±0.55	55.55±0.23	36.66±0.64		111.73±0.63		54.71±0.65		58.69±0.87	39.46±0.70		20.69±0.24		
**HDN1**		8.33±0.32		60.71±0.45	60.91±0.32	31.92±0.46		118.93±0.44		52.93±0.63		55.02±0.89	39.81±0.65		19.91±0.23		
**HDN2**		8.33±0.34		65.69±0.34	54.30±0.45	30.83±0.43		108.93±0.55		51.48±0.81		55.37±0.88	38.48±0.44		19.27±0.22		
**HDN3**		8.55±0.23		67.35±0.65	61.85±0.34	33.06±0.41		114.78±0.62		53.44±0.51		60.67±0.55	41.92±0.50		21.92±0.33		

### 3.4. Profile of fatty acids

A significant difference (P≤ 0.05) has been observed (Table 6) in the profile of fatty acids which include myristic acid (df_2_, F = 16382.91), pentadecylic acid (df_2_, F = 117.19), palmitic acid (df_2_, F = 32488.54), margaric acid (df_2_, F = 537.79), stearic acid (df_2_, F = 6992.78), tetrasenoic acid (df_2_, F = 300.16), pentadecenoic acid (df_2_, F = 56.42), palmitoleic acid (df_2_, F = 100193.06), heptadecenoic acid (df_2_, F = 185.97), oleic acid (df_2_, F = 21654.27), linoleic acid (df_2_, F = 2955.91), eicosadienoic acid (df_2_, F = 208.46), α-linolenic acid (df_2_, F = 257407.01), eicosapentanoic acid (df_2_, F = 25229.29), decosapentanoic acid (df_2_, F = 72782.66), decosahexanoic acid (df_2_, F = 104.94) among three different densities treatment.

**Table 6:** Analysis (Mean±SE) of fatty acids (Mean±SE) in total lipids extracted from muscles samples of in three density treatment (LDN (1.50 kg/m^3^), MDN (3.00 kg/m^3^), HDN (4.50 kg/m^3^)) having four N-acetyl cysteine supplementation levels (N0 = 0 mg/kg, N1 = 02 mg/kg, N2 = 04 mg/kg, N3 = 06 mg/kg). Values are expressed as percentages of total fatty acids.

**Saturated Fatty Acids (SFA)**						
**Treatment**	**Myristic Acid**	**Pentadecylic Acid**	**Palmitic Acid**	**Margaric Acid**	**Stearic Acid**	
**LDN0**	3.13±0.21	1.31±0.03	32.44±0.33	0.41±0.02	6.41±0.11	
**LDN1**	2.99±0.12	1.18±0.03	33.21±0.44	0.38±0.03	6.74±0.12	
**LDN2**	3.23±0.11	1.21±0.04	33.31±0.55	0.33±0.01	6.40±0.13	
**LDN3**	2.49±0.12	1.36±0.04	34.41±0.60	0.32±0.09	7.54±0.14	
**MDN0**	3.83±0.13	1.30±0.05	32.41±0.45	0.40±0.03	6.22±0.16	
**MDN1**	3.46±0.30	1.31±0.05	33.41±0.54	0.35±0.04	6.53±0.13	
**MDN2**	3.01±0.12	1.29±0.04	32.17±0.30	0.43±0.05	6.35±0.15	
**MDN3**	2.98±0.23	1.20±0.06	33.11±0.43	0.33±0.03	6.79±0.17	
**HDN0**	3.35±0.32	1.18±0.06	33.21±0.42	0.29±0.03	6.33±0.16	
**HDN1**	2.88±0.32	1.30±0.09	32.25±0.47	0.26±0.02	7.49±0.16	
**HDN2**	3.68±0.12	1.23±0.08	32.33±0.32	0.33±0.02	6.12±0.16	
**HDN3**	3.79±0.23	1.23±0.07	33.11±0.40	0.30±0.03	6.21±0.19	
**Monounsaturated Fatty Acids (MUFA)**						
**Treatment**	**Tetrasenoic Acid**	**Pentadecenoic Acid**	**Palmitoleic Acid**	**Heptadecenoic Acid**	**Oleic Acid**	
**LDN0**	0.39±0.02	0.53±0.09	11.73±0.13	1.50±0.09	20.18±0.22	
**LDN1**	0.40±0.03	0.46±0.04	12.52±0.15	1.61±0.08	19.78±0.23	
**LDN2**	0.32±0.04	0.61±0.03	10.75±0.16	1.59±0.06	17.75±0.24	
**LDN3**	0.66±0.05	0.54±0.22	9.81±0.11	1.63±0.09	20.46±0.20	
**MDN0**	0.41±0.05	0.53±0.23	10.77±0.11	1.60±0.07	19.56±0.22	
**MDN1**	0.45±0.06	0.58±0.24	10.52±0.14	1.58±0.05	19.77±0.22	
**MDN2**	0.44±0.08	0.43±0.24	11.63±0.15	1.40±0.05	20.42±0.24	
**MDN3**	0.33±0.08	0.56±0.13	12.44±0.16	1.58±0.04	19.88±0.23	
**HDN0**	0.28±0.06	0.58±0.32	10.65±0.17	1.56±0.03	17.85±0.26	
**HDN1**	0.56±0.06	0.44±0.21	9.12±0.18	1.52±0.03	20.36±0.24	
**HDN2**	0.35±0.04	0.51±0.22	10.87±0.21	1.54±0.06	19.36±0.32	
**HDN3**	0.31±0.04	0.50±0.32	10.32±0.20	1.53±0.04	19.87±0.43	
**Polyunsaturated Fatty Acids (PUFA)**						
**Treatment**	**Linoleic Acid**	**Eicosadienoic Acid**	**α-linolenic Acid**	**Eicosapentanoic Acid**	**Decosapentanoic Acid**	**Decosahexanoic Acid**
**LDN0**	5.81±0.22	2.79±0.11	12.69±0.19	6.52±0.11	7.61±0.11	4.86±0.16
**LDN1**	6.33±0.11	2.76±0.13	12.76±0.13	6.17±0.12	7.30±0.13	4.44±0.14
**LDN2**	6.47±0.09	2.62±0.12	8.62±0.14	4.68±0.14	5.64±0.14	4.45±0.15
**LDN3**	5.80±0.15	2.57±0.12	7.58±0.15	5.67±0.15	6.47±0.15	4.51±0.15
**MDN0**	5.68±0.15	2.60±0.10	7.79±0.18	5.91±0.16	6.47±0.13	4.29±0.13
**MDN1**	5.80±0.18	2.71±0.11	8.34±0.18	5.59±0.16	6.45±0.15	5.25±0.14
**MDN2**	5.91±0.17	2.88±0.13	12.54±0.19	6.48±0.17	7.70±0.16	4.47±0.12
**MDN3**	6.22±0.19	2.46±0.15	12.54±0.16	6.09±0.16	7.39±0.13	4.41±0.11
**HDN0**	6.44±0.17	2.76±0.13	8.52±0.16	4.72±0.14	5.52±0.14	4.35±0.14
**HDN1**	5.89±0.18	2.40±0.14	8.52±0.15	5.52±0.16	6.22±0.13	4.52±0.14
**HDN2**	5.72±0.16	2.76±0.13	8.82±0.15	5.82±0.20	6.32±0.21	4.21±0.15
**HDN3**	5.82±0.18	2.60±0.12	8.92±0.14	5.62±0.15	6.02±0.11	5.24±0.16

Fatty acid profile between different levels of N-acetyl cysteine (N) supplementation in all three-density treatment also showed a significant effect (P≤ 0.05) which includes myristic acid (df_3_, F = 5800.26), pentadecylic acid (df_3,_ F = 23.12), palmitic acid (df_3,_ F = 37738.41), margaric acid (df_3,_ F = 120.89), stearic acid (df_3,_ F = 23678.91), tetrasenoic acid (df_3,_ F = 565.67), pentadecenoic acid (df_3,_ F = 113.40), palmitoleic acid (df_3,_ F = 6039.18), heptadecenoic acid (df_3,_ F = 212.88), oleic acid (df_3,_ F = 48774.09), linoleic acid (df_3,_ F = 269.01), eicosadienoic acid (df_3,_ F = 1873.99), α-linolenic acid (df_3,_ F = 5208.34), eicosapentanoic acid (df_3,_ F = 692.07), decosapentanoic acid (df_3,_ F = 725.71), decosahexanoic acid (df_3,_ F = 6419.39).

Effect of density* N-acetyl cysteine also showed a significant effect (P≤ 0.05) on myristic acid (df_6_, F = 12380.82), pentadecylic acid (df_6,_ F = 407.69), palmitic acid (df_6,_ F = 24644.96), margaric acid (df_6,_ F = 101.15), stearic acid (df_6,_ F = 14735.24), tetrasenoic acid (df_6,_ F = 1153.44), pentadecenoic acid (df_6,_ F = 343.08), palmitoleic acid (df_6,_ F = 92982.14), heptadecenoic acid (df_6,_ F = 336.07), oleic acid (df_6,_ F = 73925.95), linoleic acid (df_6,_ F = 9540.54), eicosadienoic acid (df_6,_ F = 1557.52), α-linolenic acid (df_6,_ F = 488354.46), eicosapentanoic acid (df_6,_ F = 35026.36), decosapentanoic acid (df_6,_ F = 45635.89), decosahexanoic acid (df_6,_ F = 12064.34).

### 3.5. Digestive enzymes activity

**Fig 2:**
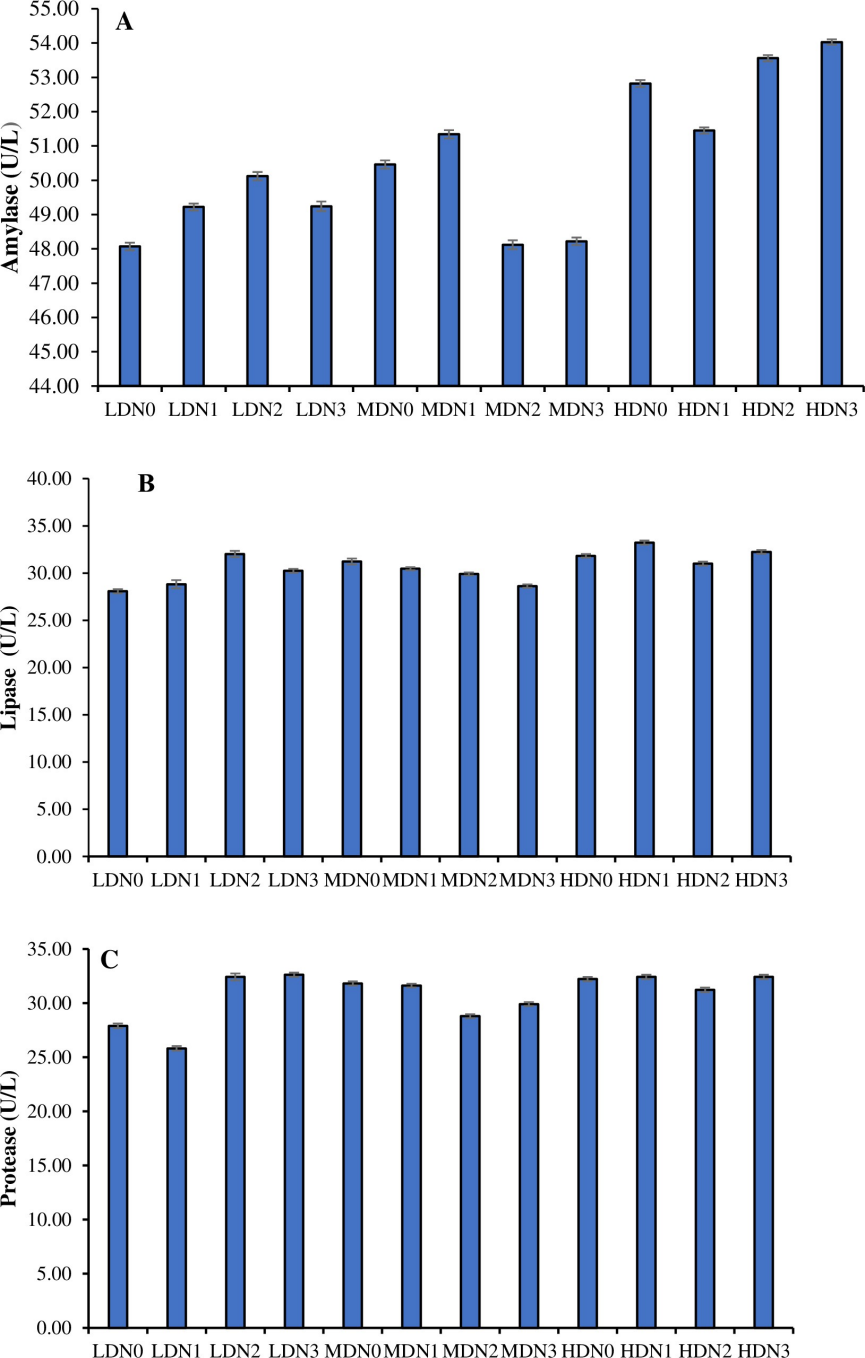
Levels of (A) amylase, (B) lipase and (C) protease (Mean ± SE) determined in three density treatments (LDN (1.50 kg/m^3^), MDN (3.00 kg/m^3^), HDN (4.50 kg/m^3^)) having four N-acetyl cysteine supplementation levels (N0 = 0 mg/kg, N1 = 02 mg/kg, N2 = 04 mg/kg, N3 = 06 mg/kg).

The activity of amylase (df_2_, F = 1230257.58), protease (df_2_, F = 407617.00) and lipase (df_2_, F = 437353.00) were significantly different (P≤ 0.05) between three density treatment (LDN, MDN, HDN) (Fig 2). Different levels of N-acetyl cysteine supplementation in density treatment (four in each treatment) showed significant variations (P≤ 0.05) in the activity of amylase (df_3_, F = 2077.25), lipase (df_3_, F = 20848.33) and protease (df_3_, F = 102063.88). In addition to this, effect of density*N-acetyl cysteine on profile of amylase (df_6_, F = 159602.91), lipase (df_6_, F = 167246.33) and protease (df_6_, F = 432818.55) was also significant (P≤ 0.05).

### 3.6. Profile of cortisol

**Fig 3:**
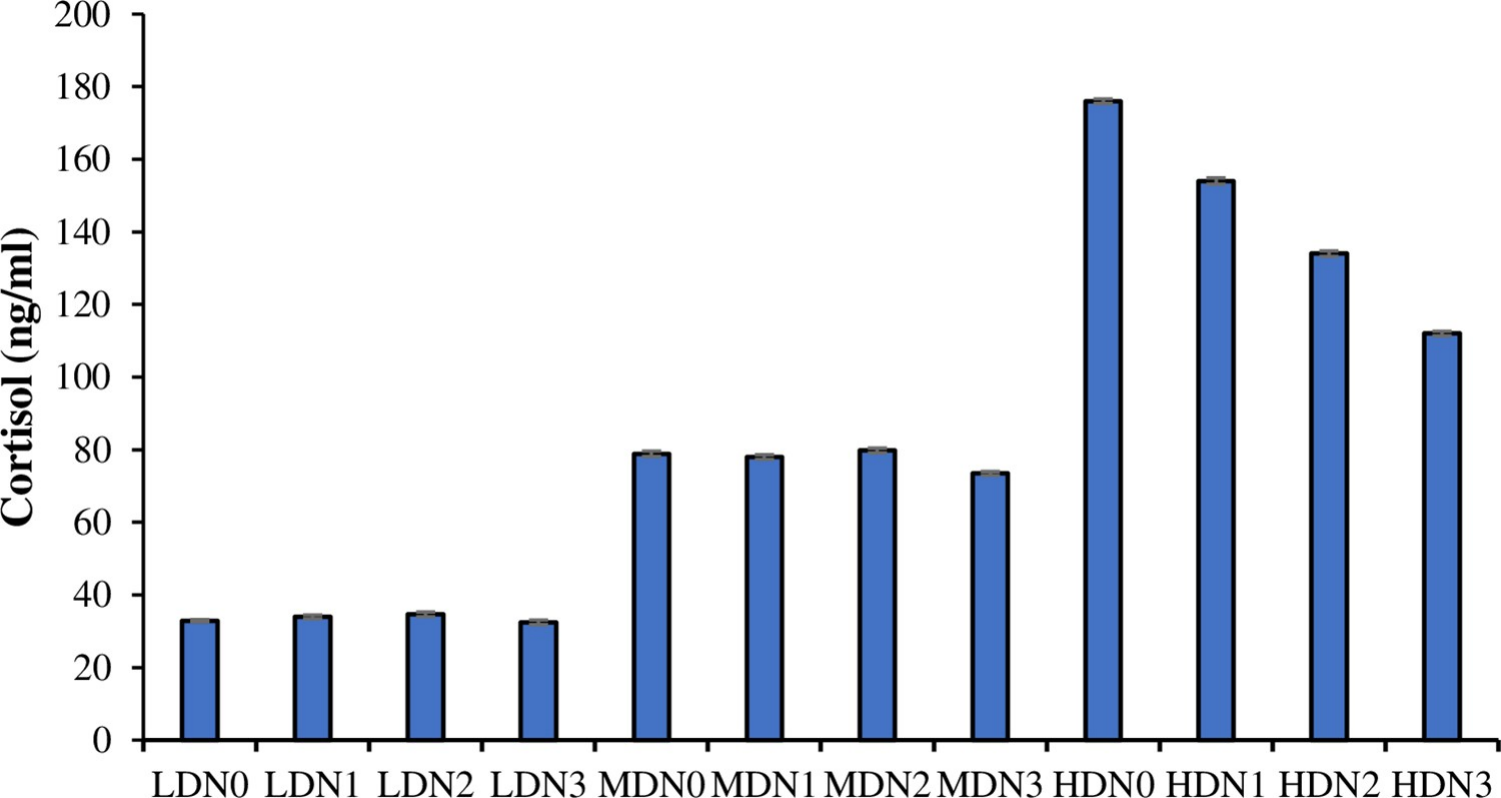
Level of cortisol (Mean ± SE) determined in three density treatments (LDN (1.50 kg/m^3^), MDN (3.00 kg/m^3^), HDN (4.50 kg/m^3^)) having four N-acetyl cysteine supplementation levels (N0 = 0 mg/kg, N1 = 02 mg/kg, N2 = 04 mg/kg, N3 = 06 mg/kg).

The levels of cortisol differed significantly (df_2_, F = 866848642.33) (P≤ 0.05) between density treatments (LDN, MDN, HDN) (Fig 3). Effect of different levels of N-acetyl cysteine supplementation in all three-density treatment (four in each treatment) was also significant (df_3_, F = 20325704.77) (P≤ 0.05). Effect of density*N-acetyl cysteine on the level of cortisol calculated by Two-way ANOVA was also significant (df_6_, F = 16375849.77) (P≤ 0.05).

### 3.7. Blood biochemistry and hematology

A significant effect (P≤ 0.05) was observed on the content of Hb (df_2_, F = 17908.33), platelets (df_2_, F = 103675833.33), WBC (df_2_, F = 4152313.08), RBC (df_2_, F = 6619.08), monocytes (df_2_, F = 2858.33), eosinophils (df_2_, F = 408.33), neutrophils (df_2_, F = 1330000.00) and lymphocytes (df_2_, F = 3260833.33) in three density treatment (Table 7). Content of triglycerides (df_2_, F = 659020833.33), ALT (df_2_, F = 248657500.00), AST (df_2_, F = 832550833.33), ALP (df_2_, F = 2342240833.33), albumin (df_2_, F = 192409.00), glucose (df_2_, F = 9630833.33), urea (df_2_, F = 39363333.33) and creatinine (df_2_, F = 4495.75) showed a significant effect (P≤ 0.05) in three density treatment (Table 8).

**Table 7:** Analysis (Mean±SE) of blood hematological (Mean ± SE) in plasma samples in three density treatments (LDN (1.50 kg/m^3^), MDN (3.00 kg/m^3^), HDN (4.50 kg/m^3^)) having four N-acetyl cysteine supplementation levels (N0 = 0 mg/kg, N1 = 02 mg/kg, N2 = 04 mg/kg, N3 = 06 mg/kg).

Treatments	Hemoglobin (g/dl)	Total RBC (× 106/μL)	Platelets (× 103/μL)	WBC (× 103/μL)	Neutrophils (%)	Lymphocytes (%)	Monocytes (%)	Eosinophils (%)
**LDN0**	7.10±0.13	1.07±0.06	144.00±0.55	34.35±0.16	86.00±0.39	14.00±0.54	2.00±0.01	2.30±0.03
**LDN1**	7.2±0.14	1.1±0.07	101.00±0.45	36.77±0.18	80.00±0.42	18.00±0.53	2.00±0.02	2.20±0.03
**LDN2**	7.4±0.12	1.09±0.05	88.00±0.43	37.77±0.17	81.00±0.40	22.00±0.45	2.00±0.02	2.30±0.03
**LDN3**	7.6±0.11	1.00±0.06	98.00±0.45	37.33±0.18	77.00±0.55	26.00±0.53	2.00±0.03	2.30±0.02
**MDN0**	7.00±0.16	1.08±0.09	123.00±0.55	33.77±0.19	71.00±0.51	17.00±0.55	2.00±0.04	2.40±0.04
**MDN1**	7.1±0.17	1.00±0.07	111.00±0.59	35.66±0.21	86.00±0.54	17.00±0.65	2.00±0.05	2.30±0.06
**MDN2**	7.3±0.16	1.03±0.09	93.00±0.69	37.87±0.23	83.00±0.65	21.00±0.76	2.00±0.04	2.10±0.06
**MDN3**	7.8±0.19	1.5±0.08	121.00±0.77	36.77±0.31	86.00±0.76	28.00±0.85	2.00±0.03	2.00±0.08
**HDN0**	6.10±0.22	0.78±0.09	165.00±0.70	25.66±0.33	84.00±0.67	16.00±0.87	2.10±0.09	2.40±0.07
**HDN1**	7.10±0.23	0.88±0.07	121.00±0.71	29.88±0.36	88.00±0.68	13.00±0.88	2.20±0.08	2.20±0.06
**HDN2**	7.00±0.23	0.98±0.06	132.00±0.73	30.44±0.34	83.00±0.69	14.00±0.69	2.20±0.07	2.10±0.05
**HDN3**	7.30±0.27	1.01±0.07	154.00±0.79	32.55±0.35	85.00±0.66	15.00±0.78	2.20±0.05	2.30±0.05

**Table 8:** Analysis on blood biochemistry (Mean ± SE) in plasma samples in plasma samples in three density treatment (LDN (1.50 kg/m^3^), MDN (3.00 kg/m^3^), HDN (4.50 kg/m^3^)) having four N-acetyl cysteine supplementation levels (N0 = 0 mg/kg, N1 = 02 mg/kg, N2 = 04 mg/kg, N3 = 06 mg/kg).

Treatments	Triglycerides (mg/dl)	Glucose (mg/dl)	ALT (U/L)	AST (U/L)	Alkaline Phosphate (U/L)	Albumin (g/dl)	Urea (mg/dl)	Creatinine (mg/dl)
**LDN0**	111.00±0.77	72.00±0.87	22.00±0.88	20.00±0.66	75.00±0.35	0.90±0.01	16.00±0.12	0.50±0.03
**LDN1**	101.00±0.87	67.00±0.89	23.00±0.76	23.00±0.62	70.00±0.31	0.80±0.03	17.00±0.14	0.50±0.04
**LDN2**	92.00±0.88	79.00±0.78	27.00±0.67	21.00±0.65	78.00±0.36	0.90±0.02	16.00±0.13	0.60±0.02
**LDN3**	89.00±0.89	83.00±0.79	24.00±0.81	25.00±0.76	88.00±0.44	0.88±0.07	18.00±0.12	0.55±0.05
**MDN0**	144.00±0.99	80.00±0.87	28.00±0.80	49.00±0.66	99.00±0.48	1.20±0.08	15.00±0.18	0.60±0.06
**MDN1**	128.00±0.88	89.00±0.85	29.00±0.78	55.00±0.76	87.00±0.55	1.00±0.09	34.00±0.16	0.77±0.05
**MDN2**	139.00±0.98	96.00±0.76	30.00±0.85	62.00±0.70	98.00±0.76	1.20±0.07	36.00±0.17	0.60±0.08
**MDN3**	132.00±0.88	81.00±0.70	32.00±0.81	69.00±0.67	100.00±0.67	1.50±0.09	40.00±0.16	0.76±0.09
**HDN0**	226.00±0.77	112.00±0.74	120.00±0.77	153.00±0.76	288.00±0.76	3.20±0.04	32.00±0.12	0.70±0.08
**HDN1**	187.00±0.80	56.00±0.73	55.00±0.74	111.00±0.73	202.00±0.78	2.00±0.05	44.00±0.15	0.78±0.09
**HDN2**	177.00±0.86	66.00±0.72	65.00±0.73	121.00±0.76	233.00±0.76	2.10±0.06	42.00±0.17	0.88±0.05
**HDN3**	188.00±0.81	78.00±0.87	73.00±0.74	133.00±0.73	255.00±0.70	2.50±0.08	43.00±0.16	0.80±0.04

Effect of different levels of N-acetyl cysteine supplementation in all three-density treatment was also studied in the hematology and biochemical parameters (four levels in each treatment). A significant effect of this supplementation was observed (P≤ 0.05) in Hb (df_3_, F = 24733.33), WBC (df_3_, F = 822619.00), RBC (df_3_, F = 1915.47), platelets (df_3_, F = 64250277.77), neutrophils (df_3_, F = 661111.11), lymphocytes (df_3_, F = 2432500.00), monocytes (df_3_, F = 58.33), eosinophils (df_3_, F = 1613.88), triglycerides (df_3_, F = 28878888.88), glucose (df_3_, F = 10614722.22), ALT (df_3_, F = 16940000.00), AST (df_3_, F = 7070000.00), ALP (df_3_, F = 47621388.88), albumin (df_3_, F = 10549.00), urea (df_3_, F = 6834722.22) and creatinine (df_3_, F = 471.33).

A significant effect (P≤ 0.05) of density*N-acetyl cysteine calculated by Two-way ANOVA were observed in the content of Hb (df_6_, F = 3675.00), WBC (df_6_, F = 68984.41), RBC (df_6_, F = 1277.30), platelets (df_6_, F = 10093611.11), neutrophils (df_6_, F = 2107777.77), lymphocytes (df_6_, F = 717500.00), monocytes (df_6_, F = 58.33), eosinophils (df_6_, F = 1030.55), triglycerides (df_6_, F = 7079722.22), glucose (df_6_, F = 19259722.22), ALT (df_6_, F = 20807500.00), AST (df_6_, F = 10680833.33), ALP (df_6_, F = 25466388.88), albumin (df_6_, F = 6675.66), urea (df_6_, F = 2022222.22) and creatinine (df_6_, F = 354.08).

### 3.8. Antioxidant assay

A significant difference (P≤ 0.05) was observed in the levels of CAT (df_2_, F = 1008147.00), SOD (df_2_, F = 9452.33), GPx (df_2_, F = 1022707.00) and MDA (df_2_, F = 3213.58) between three different density treatment (Table 9). A significant variation (P≤ 0.05) in the levels of CAT (df_3_, F = 230838.22), SOD (df_3_, F = 194717.25), GPx (df_3_, F = 902578.44) and MDA (df_3_, F = 853.02) were observed between different levels of N-acetyl cysteine supplementation in three density treatment (four in each treatment). Effect of density*N-acetyl cysteine on the levels of CAT (df_6_, F = 74889.88), SOD (df_6_, F = 6759.66), GPx (df_6_, F = 474390.77) and MDA (df_6_, F = 510.02) was significant (P≤ 0.05).

**Table 9:** Effect on antioxidant activity (Mean±SE) including catalase, superoxide dismutase and glutathione peroxidase and malondialdehyde in plasma samples in three density treatments (LDN (1.50 kg/m^3^), MDN (3.00 kg/m^3^), HDN (4.50 kg/m^3^)) having four N-acetyl cysteine supplementation levels (N0 = 0 mg/kg, N1 = 02 mg/kg, N2 = 04 mg/kg, N3 = 06 mg/kg).

Treatments	Catalase	SOD	GPX	MDA
	(U/ml)	(ng/ml)	(IU/ml)	(nmol/ml)
**LDN0**	13.9±0.10	2.05±0.08	24.80±0.21	0.26±0.02
**LDN1**	12.88±0.11	0.79±0.07	24.33±0.22	0.24±0.06
**LDN2**	13.22±0.12	0.68±0.06	24.00±0.26	0.19±0.07
**LDN3**	14.01±0.16	0.79±0.08	23.33±0.33	0.22±0.06
**MDN0**	14.9±0.17	2.91±0.09	25.01±0.32	0.33±0.05
**MDN1**	11.00±0.15	1.07±0.06	25.60±0.31	0.29±0.06
**MDN2**	10.99±0.13	0.65±0.08	23.44±0.28	0.30±0.05
**MDN3**	11.33±0.15	1.00±0.08	23.99±0.26	0.32±0.06
**HDN0**	17.30±0.17	3.22±0.07	34.44±0.28	0.67±0.04
**HDN1**	15.66±0.16	0.92±0.08	22.44±0.25	0.39±0.07
**HDN2**	14.33±0.15	0.76±0.09	25.77±0.27	0.30±0.05
**HDN3**	15.99±0.14	0.63±0.08	27.88±0.33	0.40±0.06

### 3.9. Gene expression

**Fig 4:**
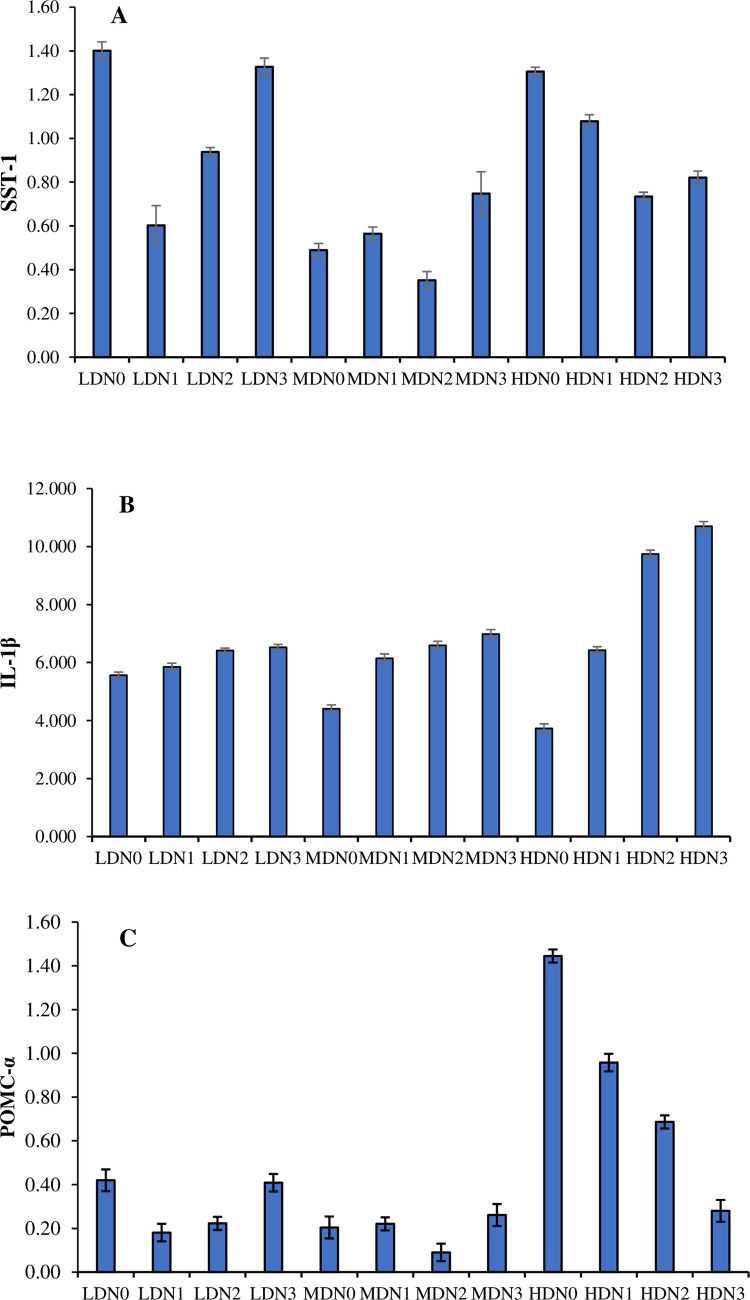
Levels of gene expression of (A) Somatostatin 1, (B) interleukin 1-β and (C) POMC-α (Mean ± SE) determined in three density treatments (LDN (1.50 kg/m^3^), MDN (3.00 kg/m^3^), HDN (4.50 kg/m^3^)) having four N-acetyl cysteine supplementation levels (N0 = 0 mg/kg, N1 = 02 mg/kg, N2 = 04 mg/kg, N3 = 06 mg/kg).

The expression of SST-1 gene (df_2_, F = 22548.57) and POMC-α (df_2_, F = 33412.45) and interleukin 1-β (df_2_, F = 235921.81) was significantly different (P≤ 0.05) between three density treatment (Fig 4). Different levels of N-acetyl cysteine supplementation in three density (four in each density treatment) showed in significant effect (P≤ 0.05) on the levels of SST-1 (df_3_, F = 6983.45), interleukin 1-β (df_3_, F = 524092.97), and POMC-α (df_3_, F = 6171.00). Effect of density*N-acetyl cysteine calculated by Two-way ANOVA on the levels of SST-1 (df_6_, F = 4582.44), interleukin 1-β (df_6_, F = 147611.67) and POMC-α (df_6_, F = 5951.00) was also significant (P≤ 0.05).

### 3.10. Histological analysis

**Fig 5:**
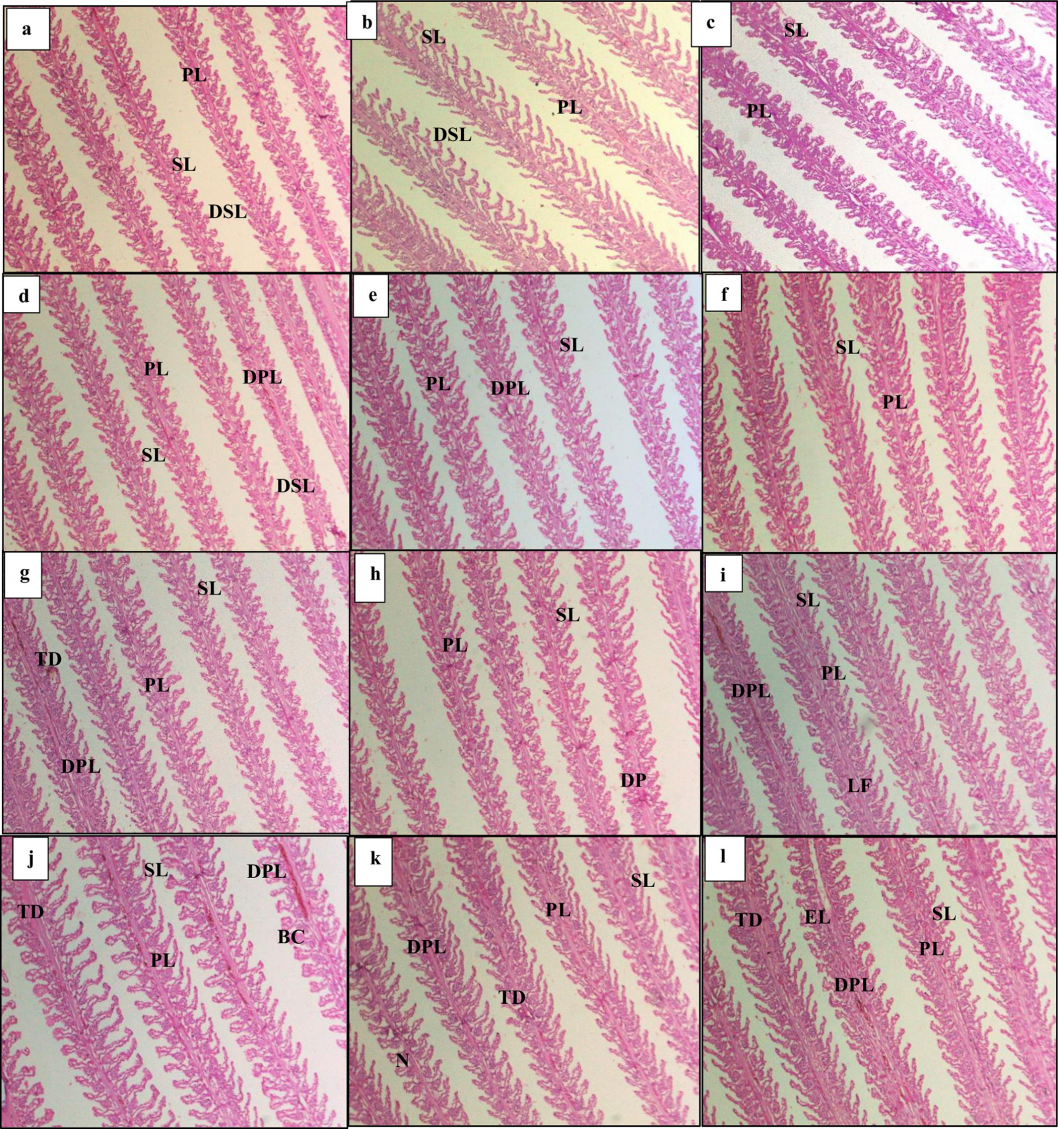
Histological changes in gills. Light micrographs of a paraffin section stained with eosin (10x). A(LDNO), B(LDN1), C(LDN2), D(LDN3), E(MDN0), F(MDN1), G(MDN2), H(MDN3), I(HDNO), J(HDN1), K(HDN2), L(HDN3). PL: Primary lamellae; SL: Secondary lamellae; DPL: Degeneration of primary lamellae; DSL: Degeneration of secondary lamellae; TD: Tissue debris; BC: Blood congestion; N: necrosis; EL: epithelial lifting; LF: Lamellar fusion.

Histology of gills was done for all treatments (density*N-acetyl cysteine) (Fig 5). Low density treatment showed minute disruption in structure of lamellae (Fig 5A and 5B). Medium and high-density treatment showed high alteration in gills structure indicated by the degeneration of primary and secondary lamella and tissue debris (Fig 5F, 5I–5L), as compared with low density treatment. In high density treatment lamellar fusion (Fig 5I), necrosis (Fig 5K), epithelial lifting (Fig 5L) (detachment of epithelial cells from secondary lamellae and congestion in blood (Fig 5J) were observed. Low density treatment showed normal structure of gills including primary lamella and secondary lamella with less or no structural alterations.

## 4. Discussion

Numerous studies have underscored the detrimental impact of overcrowding on fish physiology and overall welfare [7–9]. However, this stress can be alleviated by incorporating various dietary supplements into fish diets for enhancing their antioxidant activity and sustaining optimal growth and health. The present study investigated that the inclusion of N-acetyl cysteine as a dietary supplement resulted in a significant increase in growth across all treatments characterized by varying stocking densities. Specifically, under high stocking density (HD = 4.50 kg/m^3^), the better condition factor was observed in N2 treatment (N2 = 4 mg/kg), as compared to others. This study marks the initial exploration of the effects of N-acetyl cysteine under varying level of stress caused by stocking density. The present study coincides with previous research conducted on various fish species like Nile tilapia [41] and grass carp [42] in response to N-acetyl cysteine. This increase in growth performance might be due to supplementation of N-acetyl cysteine which lies in its unique ability to conserve methionine and promote overall fish health. Cystine being solely metabolically derived from the precursor methionine, can spare a portion of the methionine requirement in diets, thereby supporting maximum growth [43, 44].

The growth performance of fish is also associated with the SST-1. The present study observed an equivalent expression of the SST-I gene in low and high stocking density treatments although the difference in their stocking densities. This observation is consistent with previous research showing upregulation of SST-1 in tilapia under high density conditions [45], as well as in cichlids [46, 47]. Following the dietary supplementation of N-acetyl cysteine in the present study under high density conditions, the expression of SST-1 decreased, indicating a stimulatory effect on growth. There is no reported data on the impact of NAC on SST-1 gene expression. Thus, no comparative analysis has been performed.

In addition to growth parameters the chemical composition particularly crude protein and amino acid experienced a slight elevation with the inclusion of N-acetyl cysteine in the diet across various stocking densities. Similar increments in chemical composition due to N-acetyl cysteine supplementation has been previously documented in tilapia as well as in grass carp [41, 43]. No previous literature has been reported on the significance of N- acetyl cysteine against oxidative stress caused by high stocking density. Essential amino acids must be obtained from the diet, while nonessential amino acids can be synthesized by the body. Recent research has shown that some nonessential amino acids, such as glycine and cysteine, are crucial in regulating metabolic and signaling pathways, nutrient metabolism and intracellular protein turnover [48, 49]. Previous study on tilapia indicated no statistically difference among amino acid profile of tilapia [41] and grass carp [42]. No previous data has been reported on the content of fatty acid against NAC supplementation. In present study the content of fatty acid showed significant effect against all dose of NAC supplementation.

The present study revealed a significant enhancement in the activity of lipase, protease, and amylase in the intestine with the utilization of dietary supplementation of N-acetyl cysteine. Moreover, cysteine has the potential to enhance growth rates by improving the digestibility of protein and fat. The enhanced activity of digestive enzymes promotes the optimal nutrient absorption and digestion [50]. Previous study on rainbow trout indicates a significant increase in the activity of amylase against NAC supplementation [56] while in carp no effect has been observed in the activity of amylase lipase and protease [61]. In present study, fish subjected to high-density conditions and fed with no supplementation of N acetyl cysteine exhibited elevated cortisol levels, indicative of increased stress levels. These changes might be due to alterations in catecholamine levels, corticosteroid hormone levels. Stress level was also due to the shifts in the blood profiles of fish, as documented in previous research in contrast to lower density treatments [51]. The present study suggests a reduction in cortisol levels under high-density conditions with the most significant effect observed with N-acetyl cysteine treatment, particularly at the N3 dosage.

The assessment of stress levels was also conducted by analyzing the gene expression of POMC-α. It was observed that at high density the expression of this gene was elevated indicating high stress. However, following N-acetyl cysteine supplementation particularly in the N3 treatment stress levels notably decreased. The increased expression of POMC-α is linked to escalated stress levels triggering the activation of the hypothalamic-pituitary-adrenal (HPA) axis [52]. This cascade involves the release of corticotropin-releasing factor (CRF) which in turn stimulates the synthesis of pituitary pro-opiomelanocortin (POMC). Subsequently, POMC undergoes processing into adrenocorticotropic hormone (ACTH) ultimately leading to the stimulation of cortisol release via the melanocortin 2 receptor (MC2R) [53].

Dietary supplementation of N-acetyl cysteine improved the immune response of fish under different stocking densities. In the present study high level of red blood cells (RBC) and hemoglobin was detected in fish reared under high-density conditions following dietary supplementation with N-acetyl cysteine (N3 = 6mg/kg), in contrast to the N0 treatment. Red blood cells (RBC) and hemoglobin play a crucial role in oxygen transport to fish tissues and the removal of detrimental substances through the gills into surrounding environment [54]. This finding is consistent with similar study in rainbow trout [55]. The triglyceride levels were noted to be lower in fish reared at high density and supplemented with N-acetyl cysteine in their diet compared to the N0 treatment which aligns with [55]. This reduction may be attributed to enhanced lipid metabolism, potentially promoting lipolysis and reducing fat accumulation in the fish. The count of white blood cells (WBC), which serve as the primary defense mechanism in fish, tends to rise in reaction to infections or any stress [56]. In this study, an augmentation in WBC count was observed with the dietary addition of N-acetyl cysteine under high-density conditions, consistent with earlier investigations [55]. AST and ALT are gluconeogenic enzymes primarily synthesized in the liver. Elevated levels of AST and ALT have been proposed to be influenced by membrane permeability, leading to the direct release of these enzymes into the bloodstream. The increase in serum ALT activity may be attributed to liver tissue damage resulting from any type of stress [57]. The peak levels of ALT and AST under high-density conditions were noted in the N0 treatment, suggesting possible liver cell damage. Conversely, the levels of these enzymes decreased in treatments supplemented with N-acetyl cysteine, consistent with previous findings in [41, 55]. Contrary to the current study the level of ALT, AST were increased against NAC in carp [61]. Assessing the immune response of fish which were linked to their ability to neutralize harmful free radicals and reactive oxygen species (ROS) through antioxidant mechanisms. These defenses act as safeguards against stress-induced damage to tissues and organs. Two key antioxidant enzymes, superoxide dismutase (SOD) and catalase (CAT), are essential in regulating ROS levels to maintain a healthy balance [58]. The presence of oxidative enzymes such as CAT, SOD, and GPx helps to mitigate the negative impacts of high density when fish are supplemented with dietary N-acetyl cysteine (N3) in contrast to the N0 treatment. Comparable outcomes have been observed across different fish species [41, 42, 55, 59–61]. Additionally, MDA serves as an indicator of oxidative stress associated with lipid peroxidation, which escalates under conditions of increased oxidative stress. However, in present study the level of MDA declined after N-acetyl cysteine supplement. The present results consistent with previous studies in Nile tilapia [41], grass carp [42], rainbow trout [55, 59, 60], common carp [61].

The antioxidant system and immune response in fish are positively correlated contributing to their overall well-being. The immune response of fish was evaluated at the genetic level by assessing the expression of the IL-1β gene. IL-1β an interleukin released by activated macrophages which plays a critical role in regulating innate immune functions and inflammatory responses. The present study indicates that supplementation with N-acetyl cysteine resulted in an increase in the expression of the IL-1β gene compared to the N0 treatment, thereby enhancing the immune system. The most favorable outcomes were observed with the N3 dose. Limited data exist regarding the gene expression of IL-1β in response to N-acetyl cysteine supplementation, but similar patterns were observed in tilapia [41].

## 5. Conclusion

The current investigation has determined that incorporating N-acetyl cysteine into the diet across different stocking density treatments leads to improved growth, better antioxidant response and notable regulation of stress-related genes such as POMC-α. N-acetyl cysteine emerges as a potent dietary supplement for mitigating oxidative stress as evidenced by enhancements in activity of antioxidant biomarkers and expression of POMC-α and IL-1β genes. While all doses of N-acetyl cysteine exhibited efficacy in alleviating oxidative stress parameters, the N3 dose (6mg/kg) yielded the most favorable outcomes, notably in terms of boosted antioxidant enzyme levels, IL-1β level and suppressed POMC-α gene expression particularly against high density conditions (4.50 kg/m^3^). This study underscores the potential of N-acetyl cysteine to enhance the health of tilapia under conditions of high stocking density. Incorporating dietary supplementation of N-acetyl cysteine at a dosage of 6mg/kg could prove beneficial in intensive farming setups. Intensive farming in which high stocking densities are employed, enabling increased yield without compromising the well-being of the fish.

## Supporting information

S1 File All data have been provided in the supporting information. (XLSX)

## References

[1] Yue GH, Lin HR, Li JL. Tilapia is the fish for next-generation aquaculture. Int J Mar Sci Ocean Technol. 2016;3(1):11–3.

[2] Agriculture Organization of the United Nations. Fisheries Department. The State of World Fisheries and Aquaculture, FAO.; 2020.

[3] Andrade T, Afonso A, Pérez-Jiménez A, Oliva-Teles A, de las Heras V, Mancera JM, et al. Evaluation of different stocking densities in a Senegalese sole (*Solea senegalensis*) farm: implications for growth, humoral immune parameters and oxidative status. Aquac. 2015;438:6–11. 10.1016/j.aquaculture.2014.12.034.

[4] Tolussi CE, Hilsdorf AW, Caneppele D, Moreira RG. The effects of stocking density in physiological parameters and growth of the endangered teleost species piabanha, *Brycon insignis* (Steindachner, 1877). Aquac. 2010;310(1–2):221–8. 10.1016/j.aquaculture.2010.10.007

[5] Best J, Nijhout HF, Reed M. Serotonin synthesis, release and reuptake in terminals: a mathematical model. T Biol Med. 2010;7:1–26. doi: 10.1186/1742-4682-7-34 20723248 PMC2942809

[6] Barton BA. Stress in fishes: a diversity of responses with particular reference to changes in circulating corticosteroids. Integr compar biol. 2002;42(3):517–25. doi: 10.1093/icb/42.3.517 21708747

[7] Gang L, Hongxin T, Guozhi L, Chuan SD. Effect of density on *Scortum barcoo* (McCulloch & Waite) juvenile performance in circular tanks. Aquac Res. 2010;41(12):1898–904. doi: 10.1111/j.1365-2109.2010.02556.x

[8] Yarahmadi P, Miandare HK, Hoseinifar SH, Gheysvandi N, Akbarzadeh A. The effects of stocking density on hemato-immunological and serum biochemical parameters of rainbow trout (*Oncorhynchus mykiss*). Aquac Int. 2015; 23:55–63. 10.1007/s10499-014-9797-z

[9] Da Costa OT, Dias LC, Malmann CS, de Lima Ferreira CA, do Carmo IB, Wischneski AG, et al. The effects of stocking density on the hematology, plasma protein profile and immunoglobulin production of juvenile tambaqui (*Colossoma macropomum*) farmed in Brazil. Aquac. 2019;499:260–8. 10.1016/j.aquaculture.2018.09.040

[10] Odhiambo E, Angienda PO, Okoth P, Onyango D. Stocking density induced stress on plasma cortisol and whole blood glucose concentration in Nile tilapia fish (*Oreochromis niloticus*) of lake Victoria, Kenya. Int J Zool. 2020;2020:1–8. 10.1155/2020/9395268

[11] Ahmad B, Ali A, Naz D, Raziq S, Khan A, Aziz A. Biochemical composition of fish and changes during processing and storage. Biosci Res. 2020; 18(2): 1903–1913.

[12] Evans MD, Cooke MS. Factors contributing to the outcome of oxidative damage to nucleic acids. Bioessays. 2004;26(5):533–42. doi: 10.1002/bies.20027 15112233

[13] Rius-Pérez S, Torres-Cuevas I, Millán I, Ortega ÁL, Pérez S. PGC-1α, inflammation, and oxidative stress: an integrative view in metabolism. Oxidative med cell longev. 2020;2020. 10.1155/2020/1452696 PMC7085407 32215168

[14] Saera-Vila A, Calduch-Giner JA, Prunet P, Pérez-Sánchez J. Dynamics of liver GH/IGF axis and selected stress markers in juvenile gilthead sea bream (*Sparus aurata*) exposed to acute confinement: differential stress response of growth hormone receptors. Comp Biochem Physiol Part A: Mol Integr Physiol. 2009;154(2):197–203. 10.1016/j.cbpa.2009.06.004 19524697

[15] Liu ZS, Zhang L, Chen WL, He CF, Qian XY, Liu WB, et al. Insights into the interaction between stocking density and feeding rate in fish *Megalobrama ambylcephala* based on growth performance, innate immunity, antioxidant activity, and the GH-IGF1 axis. Aquac. 2024;580:740355. 10.1016/j.aquaculture.2023.740355

[16] Yuan X, Tao L, Hu X, Lin R, Yang J, Feng M, et al. Expression profile analysis of muscle growth regulation genes and effects of water flow stress on their expression levels in zebrafish. 2023. 10.21203/rs.3.rs-3109262/v1

[17] Jomova K, Raptova R, Alomar SY, Alwasel SH, Nepovimova E, Kuca K, et al. Reactive oxygen species, toxicity, oxidative stress, and antioxidants: Chronic diseases and aging. *Arch toxicol*. 2023;97(10):2499–574. doi: 10.1007/s00204-023-03562-9 37597078 PMC10475008

[18] Chen B, Lu Y, Chen Y, Cheng J. The role of Nrf2 in oxidative stress-induced endothelial injuries. *J Endocrinol*. 2015;225(3):R83–99. doi: 10.1530/JOE-14-0662 25918130

[19] Biebermann H, Kühnen P, Kleinau G, Krude H. The neuroendocrine circuitry controlled by POMC, MSH, and AGRP. Appetite control. 2012:47–75. doi: 10.1007/978-3-642-24716-3_3 22249810

[20] Shi C, Lu Y, Zhai G, Huang J, Shang G, Lou Q, et al. Hyperandrogenism in POMCa-deficient zebrafish enhances somatic growth without increasing adiposity. J Mol Cell Biol. 2020;12(4):291–304. doi: 10.1093/jmcb/mjz053 31237951 PMC7232124

[21] Very NM, Sheridan MA. The role of somatostatins in the regulation of growth in fish. Fish Physiol Biochem. 2002; 27:217–26. 10.1023/B:FISH.0000032727.75493.e8

[22] Feng P, Tian C, Lin X, Jiang D, Shi H, Chen H, et al. Identification, Expression, and Functions of the Somatostatin Gene Family in Spotted Scat (*Scatophagus argus*). Genes. 2020;11(2):194. 10.3390/genes11020194 32059553 PMC7073721

[23] Zou J, Secombes CJ. The function of fish cytokines. Biol, 2016;5(2), 23. doi: 10.3390/biology5020023 27231948 PMC4929537

[24] Hoseinifar SH, Yousefi S, Van Doan H, Ashouri G, Gioacchini G, Maradonna F, et al. Oxidative stress and antioxidant defense in fish: the implications of probiotic, prebiotic, and synbiotics. Reviews in Fisheries Science & Aquaculture. 2020;29(2):198–217. doi: 10.1080/23308249.2020.1795616

[25] Gao J, Koshio S, Ishikawa M, Yokoyama S, Nguyen BT, Mamauag RE. Effect of dietary oxidized fish oil and vitamin C supplementation on growth performance and reduction of oxidative stress in Red Sea Bream Pagrus major. Aquac Nutr. 2013;19(1):35–44. 10.1111/j.1365-2095.2011.00921.x

[26] Küçükbay FZ, Yazlak H, Karaca I, Sahin N, Tuzcu M, Cakmak MN, et al. The effects of dietary organic or inorganic selenium in rainbow trout (*Oncorhynchus mykiss*) under crowding conditions. Aquac Nutr. 2009;15(6):569–76. 10.1111/j.1365-2095.2008.00624.x

[27] Hamid ZA, Tan HY, Chow PW, Harto KA, Chan CY, Mohamed J. The role of N-acetylcysteine supplementation on the oxidative stress levels, genotoxicity and lineage commitment potential of ex vivo murine haematopoietic stem/progenitor cells. Sultan Qaboos University Medical J. 2018;18(2):130. doi: 10.18295/squmj.2018.18.02.002 30210840 PMC6132511

[28] Zhang M, Xia H, Yu M, Zhu L, Ju L, Chen J, et al. N-acetylcysteine prevents cytotoxic effects induced by man-made mineral fibers in a human bronchial epithelial cell line. Toxicol in Vitro. 2018; 53:200–7. doi: 10.1016/j.tiv.2018.08.012 30145358

[29] De Andrade KQ, Moura FA, Dos Santos JM, De Araújo OR, de Farias Santos JC, Goulart MO. Oxidative stress and inflammation in hepatic diseases: therapeutic possibilities of N-acetylcysteine. Int J mol Sci. 2015;16(12):30269–308. doi: 10.3390/ijms161226225 26694382 PMC4691167

[30] Jokanović M. Biotransformation of organophosphorus compounds. Toxicol. 2001;166(3):139–60. doi: 10.1016/s0300-483x(01)00463-2 11543910

[31] Camera E, Picardo M. Analytical methods to investigate glutathione and related compounds in biological and pathological processes. J Chromatogr B. 2002;781(1–2):181–206. doi: 10.1016/s1570-0232(02)00618-9 12450659

[32] Bharath S, Hsu M, Kaur D, Rajagopalan S, Andersen JK. Glutathione, iron and Parkinson’s disease. Biochem pharmacol. 2002;64(5–6):1037–48. doi: 10.1016/s0006-2952(02)01174-7 12213603

[33] Hanigan MH. γ-Glutamyl transpeptidase, a glutathionase: its expression and function in carcinogenesis. Chem biol interac. 1998;111:333–42. 10.1016/S0009-2797(97)00170-1 9679564

[34] Banaclocha MM. Therapeutic potential of N-acetylcysteine in age-related mitochondrial neurodegenerative diseases. Medical hypotheses. 2001;56(4):472–7. doi: 10.1054/mehy.2000.1194 11339849

[35] Aruoma OI, Halliwell B, Hoey BM, Butler J. The antioxidant action of N-acetylcysteine: its reaction with hydrogen peroxide, hydroxyl radical, superoxide, and hypochlorous acid. Free radic biol med. 1989;6(6):593–7. doi: 10.1016/0891-5849(89)90066-x 2546864

[36] Samuni Y, Goldstein S, Dean OM, Berk M. The chemistry and biological activities of N-acetylcysteine. BBA General Subjects. 2013;1830(8):4117–29. doi: 10.1016/j.bbagen.2013.04.016 23618697

[37] Aldini G, Altomare A, Baron G, Vistoli G, Carini M, Borsani L, et al. N-Acetylcysteine as an antioxidant and disulphide breaking agent: the reasons why. Free radic research. 2018;52(7):751–62. doi: 10.1080/10715762.2018.1468564 29742938

[38] Fatima S, Komal W, Manzoor F, Latif AA, Liaqat R, Ameen S, et al. Analysis of the growth performance, stress, profile of fatty acids and amino acids and cortisol in Tilapia (*Oreochromis niloticus*), cultured at high stocking density using in-pond raceway system. Saudi J Biol Sci. 2021;28(12):7422–31. 10.1016/j.sjbs.2021.08.048 34867046 PMC8626304

[39] AOAC. Official method of Analysis. 18th Edition, Association of Officiating Analytical Chemists, Washington DC, Method 935.14 and 992.24. 2005.

[40] Folch J, Lees M, Sloane-Stanley GM. A Simple Method for the Isolation and Purification of Total Lipids from Animal Tissues. J Biol Chem. 1957; 226:497–509. 10.1016/S0021-9258(18)64849-5 13428781

[41] Xie S, Zhou W, Tian L, Niu J, Liu Y. Effect of N-acetyl cysteine and glycine supplementation on growth performance, glutathione synthesis, anti-oxidative and immune ability of Nile tilapia, *Oreochromis niloticus*. Fish shellfish immunol. 2016; 55:233–41. 10.1016/j.fsi.2016.05.033 27235905

[42] Xie S, Tian L, Niu J, Liang G, Liu Y. Effect of N-acetyl cysteine and glycine supplementation on growth performance, glutathione synthesis, and antioxidative ability of grass carp, *Ctenopharyngodon idella*. Fish physiol biochem. 2017; 43:1011–20. 10.1007/s10695-017-0348-1 28124206

[43] He JY, Han B, Tian LX, Yang HJ, Zeng SL, Liu YJ. The sparing effect of cystine on methionine at a constant TSAA level in practical diets of juvenile Nile tilapia *Oreochromis niloticus*. Aquac research. 2016;47(7):2031–9. 10.1111/are.12657

[44] Li P, Mai K, Trushenski J, Wu G. New developments in fish amino acid nutrition: towards functional and environmentally oriented aquafeeds. Amino acids. 2009;37:43–53. doi: 10.1007/s00726-008-0171-1 18751871

[45] Rodriguez‐Barreto D, Rey O, Uren‐Webster TM, Castaldo G, Consuegra S, Garcia de Leaniz C. Transcriptomic response to aquaculture intensification in Nile tilapia. Evol App. 2019;12(9):1757–71. doi: 10.1111/eva.12830 31548855 PMC6752142

[46] Hofmann HA, Fernald RD. Social status controls somatostatin neuron size and growth. *J Neurosci*. 2000;20(12):4740–4. doi: 10.1523/JNEUROSCI.20-12-04740.2000 10844043 PMC6772449

[47] Trainor BC, Hofmann HA. Somatostatin and somatostatin receptor gene expression in dominant and subordinate males of an African cichlid fish. Behav brain res. 2007;179(2):314–20. doi: 10.1016/j.bbr.2007.02.014 17374406 PMC2696992

[48] Damodaran S, Parkin KL. Amino acids, peptides, and proteins. In Fennema’s food chemistry 2017:235–356. CRC Press.

[49] Wu G. Functional amino acids in nutrition and health. Amino Acids. 2013; 45:407–411 doi: 10.1007/s00726-013-1500-6 23595206

[50] Nordrum S, Krogdahl Å, Røsjø C, Olli JJ, Holm H. Effects of methionine, cysteine and medium chain triglycerides on nutrient digestibility, absorption of amino acids along the intestinal tract and nutrient retention in Atlantic salmon (*Salmo salar* L.) under pair-feeding regime. Aquac. 2000;186(3–4):341–60. 10.1016/S0044-8486(99)00385-3

[51] Saurabh S, Sahoo PK. Lysozyme: an important defence molecule of fish innate immune system. Aquac res. 2008;39(3):223–39. 10.1111/j.1365-2109.2007.01883.x

[52] Pham LP, Jordal AE, Nguyen MV, Rønnestad I. Food intake, growth, and expression of neuropeptides regulating appetite in clown anemonefish (*Amphiprion ocellaris*) exposed to predicted climate changes. Gen Comp Endocrinol. 2021;304:113719. 10.1016/j.ygcen.2021.113719 33476660

[53] Kalananthan T, Lai F, Gomes AS, Murashita K, Handeland S, Rønnestad I. The melanocortin system in Atlantic salmon (*Salmo salar* L.) and its role in appetite control. Front neuroanat. 2020;14:48. 10.3389/fnana.2020.00048 32973463 PMC7471746

[54] Mohammadi G, Rashidian G, Hoseinifar SH, Naserabad SS, Van Doan H. Ginger (*Zingiber officinale*) extract affects growth performance, body composition, haematology, serum and mucosal immune parameters in common carp (*Cyprinus carpio*). *Fish Shellfish Immunol*. 2020;99:267–73. 10.1016/j.fsi.2020.01.032 31981777

[55] Ucar A, Özgeriş FB, Yeltekin AÇ, Parlak V, Alak G, Keleş MS, et al. The effect of N‐acetylcysteine supplementation on the oxidative stress levels, apoptosis, DNA damage, and hematopoietic effect in pesticide‐exposed fish blood. J Biochem Mol Toxicol. 2019;33(6):e22311. doi: 10.1002/jbt.22311 30801904

[56] Vahedi AH, Hasanpour M, Akrami R, Chitsaz H. Effect of dietary supplementation with ginger (*Zingiber officinale*) extract on growth, biochemical and hemato-immunological parameters in juvenile beluga (*Huso huso*). Sustainable Aquac Health Management J. 2017;3(1):26–46. http://ijaah.ir/article-1-134-en.html

[57] Kaya İ, Deveci Ha, Kaya Mm, Deveci A, Kirpik Ma, Yilmaz M. The Effects of Pesticides at Sublethal Doses on the Levels of Oxidative Stress and Biochemical Parameters in Some Economically Important Fishes. Oxidative Stress and Antioxidant Defense System. 2021:1.

[58] Senso L, Suárez MD, Ruiz-Cara T, García-Gallego M. On the possible effects of harvesting season and chilled storage on the fatty acid profile of the fillet of farmed gilthead sea bream (*Sparus aurata*). Food Chem. 2007;101(1):298–307. doi: 10.1016/j.foodchem.2006.01.036

[59] Alak G, Yeltekin AÇ, Özgeriş FB, Parlak V, Uçar A, Keleş MS, et al. Therapeutic effect of N-acetyl cysteine as an antioxidant on rainbow trout’s brain in cypermethrin toxicity. Chemosphere. 2019;221:30–6. doi: 10.1016/j.chemosphere.2018.12.196 30634146

[60] Atamanalp M, Parlak V, Özgeriş FB, Çilingir Yeltekin A, Ucar A, Keleş MS, et al. Treatment of oxidative stress, apoptosis, and DNA injury with N-acetylcysteine at simulative pesticide toxicity in fish. Toxicol Mechanisms Methods. 2021;31(3):224–34. doi: 10.1080/15376516.2021.1871794 33412942

[61] Zhu R, Shang GJ, Zhang BY, Wang HT, Li L, Wei XF, et al. Unlocking the potential of N-acetylcysteine: Improving hepatopancreas inflammation, antioxidant capacity and health in common carp (*Cyprinus carpio*) via the MAPK/NF-κB/Nrf2 signalling pathway. Fish Shellfish Immunol. 2024;144:109294. 10.1016/j.fsi.2023.109294 38092096

